# Clinical relevance of timing of assessment of ICU mortality in patients with moderate-to-severe Acute Respiratory Distress Syndrome

**DOI:** 10.1038/s41598-023-28824-5

**Published:** 2023-01-27

**Authors:** Jesús Villar, Jesús M. González-Martin, José M. Añón, Carlos Ferrando, Juan A. Soler, Fernando Mosteiro, Juan M. Mora-Ordoñez, Alfonso Ambrós, Lorena Fernández, Raquel Montiel, Anxela Vidal, Tomás Muñoz, Lina Pérez-Méndez, Pedro Rodríguez-Suárez, Cristina Fernández, Rosa L. Fernández, Tamas Szakmany, Karen E. A. Burns, Ewout W. Steyerberg, Arthur S. Slutsky

**Affiliations:** 1 CIBER de Enfermedades Respiratorias, Instituto de Salud Carlos III, 28029 Madrid, Spain; 2 Research Unit, Hospital Universitario Dr. Negrín, Barranco de La Ballena S/N, 4th Floor – South wing, 35019 Las Palmas de Gran Canaria, Spain; 3 Li Ka Shing Knowledge Institute at St. Michael’s Hospital, Toronto, ON M5B 1W8 Canada; 4 Intensive Care Unit, Hospital Universitario La Paz, IdiPaz, 28046 Madrid, Spain; 5 Surgical Intensive Care Unit, Department of Anesthesia, Hospital Clinic, IDIBAPS, 08036 Barcelona, Spain; 6 Intensive Care Unit, Hospital Universitario Virgen de Arrixaca, 30120 Murcia, Spain; 7 Intensive Care Unit, Hospital Universitario de A Coruña, 15006 La Coruña, Spain; 8 Intensive Care Unit, Hospital Universitario Regional Carlos Haya, 29010 Málaga, Spain; 9 Intensive Care Unit, Hospital General Universitario de Ciudad Real, 13005 Ciudad Real, Spain; 10 Intensive Care Unit, Hospital Universitario Río Hortega, 47012 Valladolid, Spain; 11 Intensive Care Unit, Hospital Universitario NS de Candelaria, 38010 Santa Cruz de Tenerife, Spain; 12 Intensive Care Unit, Hospital Universitario Fundación Jiménez Díaz, 28040 Madrid, Spain; 13 Intensive Care Unit, Hospital Universitario de Cruces, 48903 Barakaldo, Vizcaya Spain; 14 Research Unit, Hospital Universitario NS de Candelaria, 38010 Santa Cruz de Tenerife, Spain; 15 Thoracic Surgery, Hospital Universitario Dr. Negrín, 35019 Las Palmas de Gran Canaria, Spain; 16 Department of Intensive Care Medicine and Anesthesia, Bevan University Health Board, Newport, NP20 2UB UK; 17 Honorary Professor in Intensive Care, Cardiff University, Cardiff, CF14 4XW Wales, UK; 18 Critical Care Medicine, Unity Health Toronto-St. Michael’s Hospital, Toronto, M5B 1W8 Canada; 19 Health Research Methods, Evidence, and Impact, McMaster University, Hamilton, ON Canada; 20 Department Biomedical Data Sciences, Leiden University Medical Center, Leiden, The Netherlands; 21 Division of Critical Care Medicine, University of Toronto, Toronto, ON M5T 3A1 Canada

**Keywords:** Health care, Medical research

## Abstract

Mortality is a frequently reported outcome in clinical studies of acute respiratory distress syndrome (ARDS). However, timing of mortality assessment has not been well characterized. We aimed to identify a crossing-point between cumulative survival and death in the intensive care unit (ICU) of patients with moderate-to-severe ARDS, beyond which the number of survivors would exceed the number of deaths. We hypothesized that this intersection would occur earlier in a successful clinical trial vs. observational studies of moderate/severe ARDS and predict treatment response. We conducted an ancillary study of 1580 patients with moderate-to-severe ARDS managed with lung-protective ventilation to assess the relevance and timing of measuring ICU mortality rates at different time-points during ICU stay. First, we analyzed 1303 patients from four multicenter, observational cohorts enrolling consecutive patients with moderate/severe ARDS. We assessed cumulative ICU survival from the time of moderate/severe ARDS diagnosis to ventilatory support discontinuation within 7-days, 28-days, 60-days, and at ICU discharge. Then, we compared these findings to those of a successful randomized trial of 277 moderate/severe ARDS patients. In the observational cohorts, ICU mortality (487/1303, 37.4%) and 28-day mortality (425/1102, 38.6%) were similar (*p* = 0.549). Cumulative proportion of ICU survivors and non-survivors crossed at day-7; after day-7, the number of ICU survivors was progressively higher compared to non-survivors. Measures of oxygenation, lung mechanics, and severity scores were different between survivors and non-survivors at each point-in-time (*p* < 0.001). In the trial cohort, the cumulative proportion of survivors and non-survivors in the treatment group crossed before day-3 after diagnosis of moderate/severe ARDS. In clinical ARDS studies, 28-day mortality closely approximates and may be used as a surrogate for ICU mortality. For patients with moderate-to-severe ARDS, ICU mortality assessment within the first week of a trial might be an early predictor of treatment response.

## Introduction

Acute respiratory distress syndrome (ARDS) is a form of acute hypoxemic respiratory failure secondary to a number of predisposing insults, including sepsis, pneumonia, and traumatic injury. More than a third of mechanically ventilated patients with ARDS die in the intensive care unit (ICU)^1^. Prevention of ICU death through therapeutic interventions is a major focus of clinical research in ARDS. Despite advances in the understanding of mechanisms that lead to ARDS^2^, concerns remain regarding how and when to assess ARDS outcome, including mortality, in observational studies and randomized controlled trials (RCTs).

The selected patient populations enrolled in many RCTs might not be representative of patients managed in clinical practice. Furthermore, frequently reported outcomes may not be clinically relevant due to the way they are measured and reported^3^. Most RCTs are efficacy studies since they evaluate how an intervention works in an artificial setting that controls for multiple variables^4^. Although these trials are necessary, they may limit our ability to apply the study findings to patients in the real world. As such, reported mortality rates for ARDS patients in RCTs may not reflect those observed in clinical practice^3,5^. Among other considerations, participation in RCTs requires consent, and therefore, these trials do not include consecutive eligible patients. Moreover, most RCT patients are recruited from tertiary centers in high-income countries^1,6^.

The science behind selecting appropriate outcome measures and their optimal time of assessment in clinical studies is still immature. In general, 28-day mortality is a widely used clinical endpoint in RCTs in ARDS^7,8^. Clinicians may be better able to interpret and compare studies if a range of outcome measures were provided at selected time-points. However, it is unclear which times of ascertainment and combinations of outcome measures provide the best assessment of mortality in observational studies and therapeutic RCTs. We assume that ARDS patients want to improve and not to have their lives extended by a few days or weeks while undergoing intensive treatment at high cost^8^.

In this study, we aimed to identify a crossing-point between cumulative survival and death in the ICU of a large population of patients with moderate-to-severe ARDS treated with lung-protective mechanical ventilation (MV), beyond which the number of survivors could exceed the number of deaths, using different study designs (observational cohorts and a published therapeutic RCT).

## Methods

### Ethics approval and consent to participate

This study was approved by the Ethics Committee of Clinical Research at the Hospital Universitario Dr. Negrín (Las Palmas de Gran Canaria, Spain), and the requirement for informed consent was waived (Reference CEI/CEIm 2021-321-1) under the Royal Decree 1090/2015 of December 2015, and Royal Decree 957/2020 of November 2020 of the Spanish legislation for biomedical research based on the retrospective nature of the secondary analysis, the anonymization/dissociation of data, and with no harm and no benefit for the management of patients.

This was an ancillary study using unrestricted data from our previously published studies in patients with moderate-to-severe ARDS^1,9–14^ that were approved by the referral Ethics Committees of Hospital Universitario Dr. Negrín (Las Palmas de Gran Canaria, Spain), Hospital Virgen de La Luz (Cuenca, Spain), Hospital Clínico Universitario (Valladolid, Spain), Hospital Universitario La Paz (Madrid, Spain), and Hospital Clínico de Valencia (Valencia, Spain), and adopted by all participating centers, as required by the Spanish legislation. This study was conducted in accordance with the fundamental principles established in the Declaration of Helsinki, the Convention of the European Council related to human rights and biomedicine, the Ethical Guidelines for Health-related Research Involving Humans by the Council for International Organization of Medical Sciences (CIOMS) of the World Health Organization (WHO), and within the requirements established by the Spanish legislation for biomedical research, the protection of personal data, and bioethics. The study followed the STROBE (Strengthening the Reporting of Observational Studies in Epidemiology) guidelines^15^.

### Study design and patients

We performed an ancillary analysis of data derived from 1580 adult patients with moderate-to-severe ARDS^16^ managed with lung-protective MV in a network of ICUs under the Spanish Initiative for Epidemiology, Stratification, and Therapies of ARDS (SIESTA) (A full list of members and their affiliations appears in the Supplemental File), as previously described^1,9–13^. The current study was conducted in two steps. First, we analyzed 1303 patients included in four multicenter, observational cohort studies enrolling consecutive patients who met current criteria for moderate-to-severe ARDS^16^ including: (i) having an initiating clinical condition, (ii) developed within 1 week of a known clinical insult, or new or worsening respiratory symptoms, (iii) bilateral pulmonary infiltrates on chest imaging, (iv) no evidence of left atrial hypertension or no clinical signs of left heart failure, and (v) hypoxemia (defined by PaO_2_/FiO_2_ ≤ 200 mmHg on PEEP ≥ 5 cmH_2_O). We did not enroll mild ARDS (PaO_2_/FiO_2_ > 200 mmHg), unless these patients progressed to a more severe category during the observational periods. We excluded patients < 18 years old, with severe chronic pulmonary disease, acute cardiac failure, brain death, patients with a do-not-resuscitate orders, or postoperative patients who received MV for < 24 h. Patients were assessed at the time of moderate/severe ARDS diagnosis, and at 24 h under standardized ventilator settings (details in Supplemental File).

Subsequently, we tested the findings of our observational dataset in a cohort of 277 patients with moderate/severe ARDS enrolled in a successful (as defined by a reduction in mortality) therapeutic RCT (DEXA-ARDS) testing dexamethasone in persistent moderate/severe ARDS^14^. By design, no patients with PaO_2_/FiO_2_ > 200 mmHg at 24 h were enrolled in this trial. We selected this trial for several reasons: (i) it was an investigator-initiated clinical trial, performed in a network of 17 ICUs in teaching hospital across Spain^14^; (ii) the trial was led and coordinated by the principal investigator of the present study (JV), and thus we had detailed patient level data on all subjects in the trial; (iii) the trial is one of the very few RCTs performed in the last decade in patients with moderate/severe ARDS where overall mortality was significantly reduced in the experimental group^17^. With this new population, we studied the relevance and external validity of measuring all-cause ICU mortality as primary endpoint for future clinical trials of moderate/severe ARDS.

### Variables, outcomes, predefined rules and expectations

For the purpose of this study, we retrieved information regarding patient demographics, etiology of ARDS, Acute Physiology and Chronic Health Evaluation II (APACHE II) score^18^, arterial blood gases and MV data at moderate/severe ARDS diagnosis and at 24 h after diagnosis. Attending clinicians followed standards and recommendations for general management of critically ill patients, including antibiotic therapy, hemodynamic support, and lung-protective MV, among others (see Supplemental File). Onset of ARDS was defined as the day on which the patient met moderate-to-severe ARDS criteria. We recorded occurrence of extrapulmonary organ failures (OF), including cardiovascular system, hepatic, renal, coagulation, and central nervous system, represented in the Sequential Organ Failure Assessment (SOFA) scale^19^. Extrapulmonary OF was defined as an acute change in organ-specific SOFA score ≥ 2^20,21^. The PaO_2_/FiO_2_ and plateau pressure (Pplat) at 24 h were measured using a standardized ventilatory setting with PEEP = 10 cmH_2_O and FiO_2_ = 0.5^22,23^**,**. When patients required PEEP > 10 or FiO_2_ > 0.5, a set of rules for setting PEEP and FiO_2_ were applied only during the standardized assessment (see Supplemental File), as described and validated by our group^22,23^. Patients were followed until ICU and hospital discharge.

The primary outcome was all-cause mortality in the ICU. For the purpose of this study, outcome at selected time-points were calculated considering the patient’s dependency on ventilatory support. To determine the 7-day outcome, we calculated the cumulative number of moderate/severe ARDS patients on ventilatory support for ≤ 7 days after diagnosis who were discharged alive or dead from the ICU, independently of the day on which patients were discharged from ICU. Similarly, to determine the 28-day outcome, we calculated the cumulative number of patients on ventilatory support for ≤ 28 days who were discharged alive or dead from the ICU, independently of the day on which patients were discharged from ICU. To tabulate the 60-day outcome, we calculated the cumulative number of patients on ventilatory support for ≤ 60 days, who were discharged alive or dead from the ICU, independently of the day in which patients were discharged from ICU. No patients with extubation failure (need for reintubation or for continuing ventilatory support) were excluded from these calculations. When patients were discharged alive from the ICU, we did not take into account for the calculation of 28-day or 60-day outcome whether the clinicians, patients, or relatives [in the same hospital, in another hospital, in a nursing home, in successive hospital readmissions, or at home] considered that continuation of medical treatment was no longer meaningful, or the patient no longer consented to treatment, or the benefit of a treatment no longer outweighed its negative effects in the hospital wards. For patients discharged alive from the ICU, discharge was indicated if the patient’s vital functions were stable without life support and thus, no longer requiring ICU monitoring or treatment (if caring the patient in the hospital ward was possible). However, discharge from ICU is influenced by organizational factors (i.e. bed availability in hospital wards), individual factors (i.e. environmental characteristics to providing patient and family support), and teamwork factors (i.e. medical and nursing leadership and communication)^24^.

At each point-in-time, we analyzed differences in etiologies, oxygenation defect, lung mechanics, and severity scores between survivors and non-survivors.

### Statistical analysis

We developed an a priori statistical analysis plan. First, we separated patients into ICU survivors and non-survivors and calculated their cumulative proportion for each day on ventilatory support. Second, we analyzed baseline differences of values of selected variables between survivors and non-survivors for each time-point. Third, we required differences of mean values at a 0.005 significance level for considering a real effect size, as recently recommended^25^. Fourth, we sought to identify a crossing-point between ICU cumulative survival and cumulative death within the first 10 days of enrollment, beyond which the number of ICU survivors is expected to be higher than the number of deaths. Fifth, we hypothesized that, irrespective of the day of the crossing-point in the observational cohort, this intersection would occur earlier in a successful ARDS therapeutic trial (as defined by a reduction in mortality). Therefore, we postulated that the intersection point could be seen as predictor of treatment response (successful) or lack of response (unsuccessful). Sixth, we expected that outcome usually reported in ARDS trials (28-day mortality) would be lower than the most frequent outcome measure reported in observational studies (ICU mortality).

Values of quantitative variables are described using mean ± SD, and median and percentiles. We used the Kolmogorov–Smirnov to test normal distribution of data. We calculated frequency and percentage of qualitative variables and analyzed differences between categorical variables with Fisher´s exact test. We compared continuous variables using the Student’s *t* test, analysis of variances, Mann–Whitney U, or the Kruskall-Wallis tests, depending on their distribution and number of variables and groups. We determined the ICU mortality at each specific point-in-time of interest (7-day, 28-day, 60-day, ICU discharge), and compared main baseline variables between survivors and non-survivors at the pre-specified time periods. We determined mean differences and 95% confidence intervals (CIs) between groups. We used the R Core Team 2022 (version 4.2.1), for statistical computing (R Foundation for Statistical Computing, Vienna, Austria). For all comparisons, a two-sided *p* < 0.005 was considered a real effect size, as recommended^25^.

## Results

All-cause ICU mortality in 1303 patients was 37.4% (487/1303, 95% CI 34.8–40.0), and were similar within the four pooled cohorts (123/300, 41.0%; 114/300, 38.0%; 138/400, 34.5%; 112/303, 37.0%) (*p* = 0.366). Patient characteristics at the time of diagnosis of moderate/severe ARDS are reported in Table 1. Those cohorts included more males (vs. females), with pneumonia and sepsis being the most common etiologies of ARDS. At the time of moderate/severe ARDS diagnosis, most patients were ventilated with a tidal volume (VT) < 8 ml/kg predicted body weight, PEEP > 9 cmH_2_0, Pplat < 29 cmH_2_O, and driving pressure < 15 cmH_2_O. In general, and independently of the day of death, non-survivors were older, had more severe ARDS, higher APACHE II and SOFA scores, higher plateau and driving pressures, and higher extrapulmonary OF than survivors (Table 1). Time from initiation of MV to diagnosis of moderate/severe ARDS was similar in the observational cohort than in the trial cohort (1.29 ± 3.62 vs. 1.03 ± 2.56 days, mean difference − 0.26 days, 95%CI − 0.71–0.19; *p* = 0.256).

**Table 1:** Baseline characteristics and outcome data of an observational cohort of 1303 patients with moderate-to-severe acute respiratory distress syndrome (ARDS) at ICU discharge.

Variables	All patients (N = 1,303)	Survivors N = 816	Non-survivors N = 487	Mean difference* (95%CI)	*p*-value
Age, *years, mean* ± *SD*	57.2 ± 15.7	54.3 ± 15.7	62.0 ± 14.5	7.7 (6.0 to 9.4)	< 0.001
Sex, *n (%)*					
Male	903 (69.3)	566 (69.4)	337 (69.2)		
Female	400 (30.7)	250 (30.6)	150 (30.8)	0.2 (− 4.9 to 5.4)	0.940
Etiology*, n (%)*					
Pneumonia	590 (45.3)	378 (46.3)	212 (43.5)	2.8 (− 2.8 to 8.3)	0.326
Sepsis	364 (27.9)	201 (24.6)	163 (33.5)	8.9 (3.8 to 14.1)	< 0.001
Aspiration	141 (10.8)	90 (11.0)	51 (10.5)	0.5 (− 3.1 to 3.9)	0.779
Trauma	112 (8.6)	96 (11.8)	16 (3.3)	8.5 (5.7 to 11.2	< 0.001
Acute Pancreatitis	45 (3.5)	19 (2.3)	26 (5.3)	3.0 (0.9 to 5.5)	0.004
Multiple transfusions	13 (0.1)	9 (1.1)	4 (0.8)	0.3 (-1.1 to 1.4)	0.596
Others	38 (2.9)	23 (2.8)	15 (3.1)	0.3 (-1.5 to 2.5)	0.755
Degree of ARDS, *n (%)*					
Severe	517 (39.7)	297 (36.4)	220 (45.2)	8.8 (3.3 to 14.3)	0.002
Moderate	786 (60.3)	519 (63.6)	267 (54.8)	8.8 (3.3 to 14.3)	0.002
APACHE II score, *mean* ± *SD*	20.9 (7.0)(**§**)	19.6 ± 6.6	23.2 ± 7.1	3.6 (2.8 to 4.4)	< 0.001
SOFA score, *mean* ± *SD*	9.3 ± 3.5	8.5 ± 3.1	10.7 ± 3.7	2.2 (1.7 to 2.5)	< 0.001
PaO_2_/FiO_2_, *mmHg, mean* ± *SD*	115.7 ± 39.0	118.6 ± 38.3	110.9 ± 39.9	7.7 (3.3 to 12.1)	< 0.001
FiO_2_*, mean* ± *SD*	0.78 ± 0.19	0.78 ± 0.19	0.79 ± 0.19	0.01 (-0.01—0.03)	0.358
PaO_2_, *mmHg, mean* ± *SD*	86.0 ± 26.0	87.8 ± 26.5	82.9 ± 24.9	4.9 (2.0 to 7.8)	0.001
PaCO_2_, *mmHg, mean* ± *SD*	49.4 ± 12.9	48.6 ± 12.2	50.7 ± 13.7	2.1 (0.7 to 3.5)	0.004
pH, *mean* ± *SD*	7.30 ± 0.11	7.31 ± 0.10	7.28 ± 0.12	0.03 (0.02 to 0.04)	< 0.001
VT, *mL/kg PBW, mean* ± *SD*	6.8 ± 1.1	6.8 ± 1.0	6.8 ± 1.1	0 (− 0.1 to 0.1)	1
Respiratory rate, *mean* ± *SD*	21.6 ± 4.9	21.5 ± 4.7	21.8 ± 5.2	0.3 (-0.2 to 0.8)	0.285
Minute ventilation, *L/min, mean* ± *SD*	9.2 ± 2.2	9.2 ± 2.1	9.1 ± 2.3	0.1 (− 0.3 to 0.1)	0.423
PEEP, cmH_2_O, *mean* ± *SD*	11.8 ± 3.3	11.8 ± 3.3	11.7 ± 3.2	0.1 (− 0.5 to 0.3)	0.593
Plateau pressure, *cmH*_*2*_*O, mean* ± *SD*	26.2 ± 4.9 (**¶**)	25.6 ± 4.8	27.2 ± 4.9	1.6 (1.1 to 2.1)	< 0.001
Driving pressure, *cmH*_*2*_*O, mean* ± *SD*	14.5 ± 4.8	13.8 ± 4.5	15.5 ± 5.1	1,7 (1.2 to 2.2)	< 0.001
No. extrapulmonary OF, *mean* ± *SD*	1.7 ± 1.1	1.5 ± 1.0	2.1 ± 1.2	0.6 (0.5 to 0.7)	< 0.001
Days ventilatory support from diagnosis of moderate/severe ARDS, *mean* ± *SD*	16.7 ± 17.0	18.1 ± 17.2	14.3 ± 16.4	3.8 (1.9 to 5.7)	< 0.001
Days from initiation ventilatory support to diagnosis of moderate/severe ARDS, *mean* ± *SD*	1.3 ± 3.6	1.1 ± 2.8	1.6 ± 4.6	0.5 (0.1 to 0.9)	0.015

(*) Mean difference represents the difference between values in survivors and non-survivors.

APACHE, acute physiology and chronic health evaluation; ARDS, acute respiratory distress syndrome; CI, confidence interval; FiO_2_, fraction of inspired oxygen concentration; ICU, intensive care unit; OF, organ failure; PBW, predicted body weight; PEEP, positive end-expiratory pressure; SD, standard deviation; SOFA, sequential organ failure assessment scale; VT, tidal volume.

(§) APACHE II was missing in 11 survivors and 8 non-survivors.

(¶) Plateau pressure was missing in 6 survivors and 9 non-survivors.

**Figure 1:**
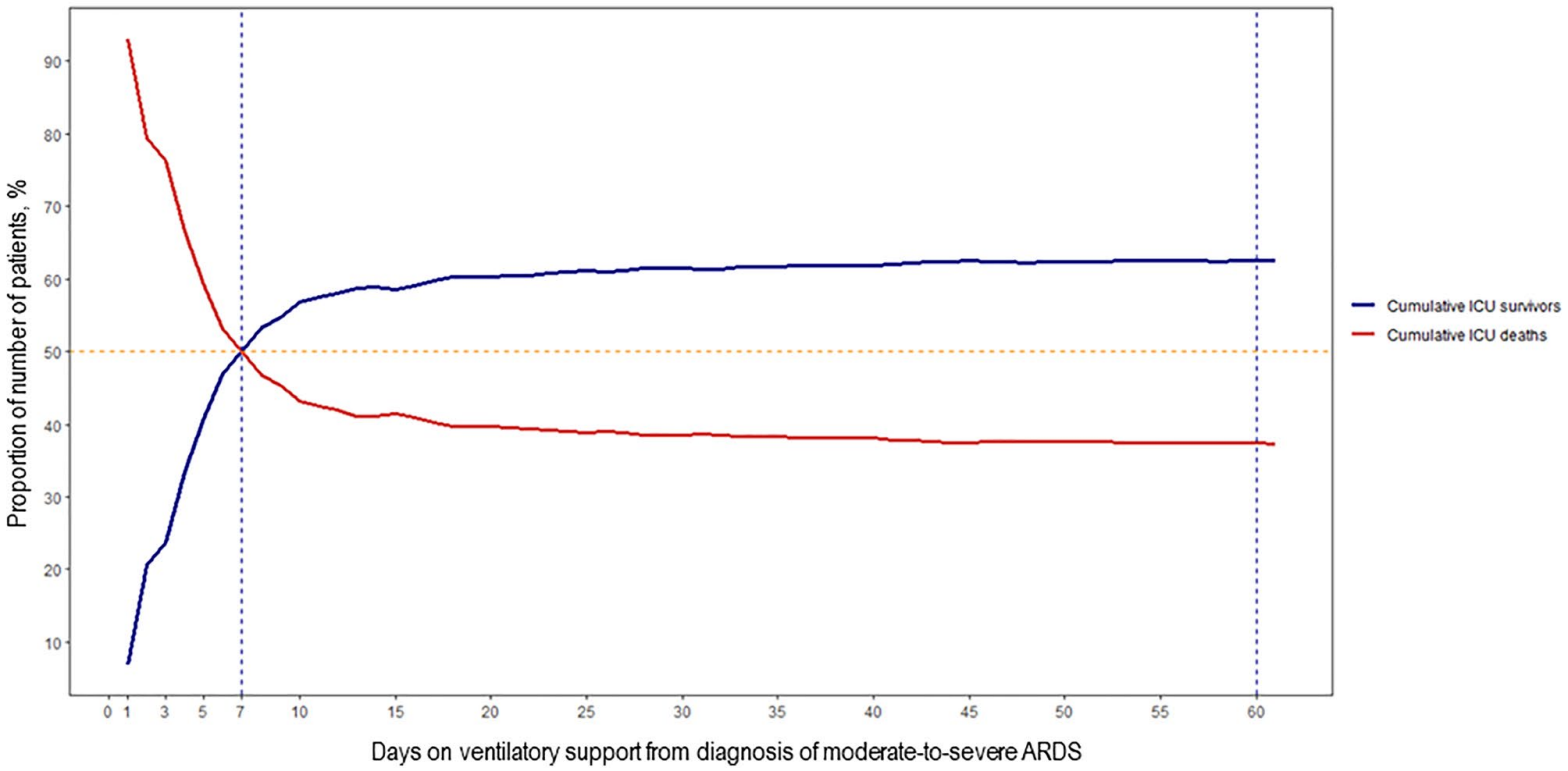
Percentage of cumulative number of survivors and non-survivors in 1303 patients with moderate-to-severe ARDS. Each day represents the cumulative percentage of patients that were ventilated up to that day and were discharged alive or dead from the intensive care unit (ICU), independently of the day on which patients were discharged from ICU (see Supplementary Table S1 for details). *Note: from 58 patients on mechanical ventilation for 1 day, 54 patients (93.1%) died in the ICU.*

The cumulative proportion of ICU survivors and non-survivors in 1303 patients of the observational dataset crossed at day-7 (Table S1, Fig. 1). A total of 412 patients were on ventilatory support for ≤ 7 days and half of these patients (n = 206) died in the ICU, representing a cumulative 7-day mortality of 50% (206/412) and an overall ICU mortality of 15.8% (206/1303) (Table 2). After day-7, the number of ICU survivors was progressively higher than non-survivors (Table S1). All-cause 28-day cumulative ICU mortality (425/1102, 38.6%) was similar to cumulative 60-day (475/1269, 37.4%) or ICU mortality (487/1303, 37.4%; difference 1.2%, 95% CI − 2.7 to 5.1, *p* = 0.549) with no relevant changes from day-28 to ICU discharge (Fig. 1). Mean baseline values of oxygenation, lung mechanics, and severity scores were different between ICU survivors and non-survivors (*p* < 0.001) at each point-in-time (Tables 1, 2, 3, Table S2). Of interest, 7-day, 28-day, 60-day, and ICU discharge mortality rates were similar across the four cohorts (Table S3). When the 1303 patients with moderate/severe ARDS were assessed at 24 h under standardized ventilatory settings, approximately 20% of patients (n = 255) had a PaO_2_/FiO_2_ > 200 mmHg (Table S4).

**Table 2:** Baseline characteristics and outcome data of 412 patients with moderate-to-severe acute respiratory distress syndrome. Number of patients represents the cumulative number of patients on ventilatory support for ≤ **7 days** and discharged dead or alive from ICU.

Variables	Patients N = 412	survivors N = 206	Non-survivors N = 206	Mean difference*(95% CI)	*p*-value
Age, *years, mean* ± *SD*	58.4 ± 15.7	54.4 ± 16.4	62.4 ± 14.0	8 (5.1 to 11.0)	< 0.001
Sex, *n (%)*					
Male	274 (66.5)	134 (65.0)	140 (68.0)		
Female	138 (33.5)	72 (35.0)	66 (32.0)	3.0 (− 6.1 to 12.0)	0.52
Etiology, *n (%)*					
Pneumonia	166 (40.3)	85 (41.3)	81 (39.3)	2.0 (− 7.4 to 11.4)	0.679
Sepsis	141 (34.2)	63 (30.6)	78 (37.9)	7.3 (− 1.9 to 16.3)	0.119
Aspiration	40 (9.7)	23 (11.2)	17 (8.3)	2.9 (− 2.9 to 8.8)	0.322
Trauma	26 (6.3)	18 (8.7)	8 (3.9)	4.8 (0.03 to 9.8)	0.045
Acute Pancreatitis	23 (5.6)	6 (2.9)	17 (8.3)	5.4 (0.9 to 10.2)	0.017
Multiple transfusions	3 (0.7)	1 (0.5)	2 (1.0)	0.5 (− 1.8 to 3.1)	0.557
Others	13 (3.2)	10 (4.9)	3 (1.5)	3.4 (− 0.1 to 7.4)	0.05
Degree of ARDS severity, *n (%)*					
Severe	152 (36.9)	58 (28.2)	94 (45.6)	17.4 (8.1 to 26.3)	< 0.001
Moderate	260 (63.1)	148 (71.8)	112 (54.4)	17.4 (8.1 to 26.3)	< 0.001
APACHE II score, *mean* ± *SD*	21.9 ± 7.9 (**§**)	18.7 ± 6.3	25.2 ± 7.9	6.5 (5.1 to 7.9)	< 0.001
SOFA score, *mean* ± *SD*	9.7 ± 3.9	7.8 ± 2.9	11.7 ± 3.7	3.9 (3.3 to 4.5)	< 0.001
PaO_2_/FiO_2_, *mmHg, mean* ± *SD*	119.6 ± 41.8	128.0 ± 38.6	111.3 ± 43.3	16.7 (8.8 to 24.6)	< 0.001
FiO_2_, *mean* ± *SD*	0.77 ± 0.19	0.74 ± 0.19	0.80 ± 0.20	0.06 (0.02 to 0.10)	0.002
PaO_2_, *mmHg, mean* ± *SD*	87.0 ± 25.9	90.9 ± 26.0	83.1 ± 25.2	7.8 (2.8 to 12.8)	0.002
PaCO_2_, *mmHg, mean* ± *SD*	48.8 ± 12.8	47.3 ± 11.7	50.2 ± 13.7	2.9 (0.4 to 5.4)	0.021
pH, *mean* ± *SD*	7.29 ± 0.12	7.32 ± 0.11	7.25 ± 0.12	0.07 (0.04 to 0.09)	< 0.001
VT, *mL/kg PBW, mean* ± *SD*	6.9 ± 1.1	6.8 ± 1.0	6.9 ± 1.1	0.1 (-0.1 to 0.3)	0.335
Respiratory rate, *mean* ± *SD*	21.5 ± 4.9	21.1 ± 4.7	21.8 ± 5.0	0.7 (-0.2 to 1.6)	0.144
Minute ventilation, *L/min, mean* ± *SD*	9.1 ± 2.2	8.9 ± 2.1	9.3 ± 2.3	0.4 (-0.03 to 0.83)	0.066
PEEP, cmH_2_O, mean ± SD	11.5 ± 3.2	11.2 ± 3.2	11.8 ± 3.3	0.4 (-0.03 to 1.23)	0.062
Plateau pressure, *cmH*_*2*_*O, mean* ± *SD*	26.1 ± 4.8 (**¶**)	24.9 ± 4.8	27.2 ± 4.5	2.3 (1.4 to 3.2)	< 0.001
Driving pressure, *cmH*_*2*_*O, mean* ± *SD*	14.6 ± 4.8	13.8 ± 4.4	15.4 ± 5.0	1.6 (0.7 to 2.5)	< 0.001
No. extrapulmonary OF, mean ± SD	1.8 ± 1.2	1.3 ± 0.9	2.4 ± 1.2	1.1 (0.9 to 1.3)	< 0.001
Days ventilatory support from diagnosis of moderate/severe ARDS, *mean* ± *SD*	4.0 ± 2.1	4.9 ± 1.7	3.2 ± 2.0	1.7 (1.3 to 2.1)	< 0.001
All-cause ICU mortality, n (%)	206 (50.0)	–	–	–	–
All-cause hospital mortality, n (%)	219 (53.2)	–	–	3.2 (-3.6 to 10.0)	0.358

(*) Mean difference represents the difference between values in survivors and non-survivors.

APACHE, acute physiology and chronic health evaluation; ARDS, acute respiratory distress syndrome; CI, confidence interval; FiO_2_, fraction of inspired oxygen concentration; ICU, intensive care unit; OF, organ failure; PBW, predicted body weight; PEEP, positive end-expiratory pressure; SD, standard deviation; SOFA, sequential organ failure assessment scale; VT, tidal volume.

(§) APACHE II was missing in 4 survivors and 4 non-survivors.

(¶) Plateau pressure was missing in 1 non-survivor.

**Table 3:** Baseline characteristics and outcome of 1,102 patients with moderate-to-severe acute respiratory distress syndrome. Number of patients represents the cumulative number of patients on ventilatory support for ≤ 28 days and discharged dead or alive from the ICU.

Variables	All patients N = 1102	survivors N = 677	Non-survivors N = 425	Mean difference* (95% CI)	*p*-value
Age, years, *mean* ± *SD*	57.0 ± 16.0	53.8 ± 16.1	62.0 ± 14.7	8.2 (6.3 to 10.1)	< 0.001
Sex, n (%)					
Male	753 (68.3)	457 (67.5)	296 (69.7)		
Female	349 (31.7)	220 (32.5)	129 (30.4)	2.1 (− 3.6 to 7.6)	0.466
Etiology, n (%)					
Pneumonia	490 (44.5)	310 (45.8)	180 (42.4)	3.4 (− 2.6 to9.4)	0.269
Sepsis	321 (29.1)	171 (25.3)	150 (35.3)	10.0 (4.4 to 15.6)	< 0.001
Aspiration	120 (10.9)	75 (11.1)	45 (10.6)	0.5 (− 3.4 to 4.2)	0.796
Trauma	95 (8.6)	81 (12.0)	14 (3.3)	8.7 (5.6 to 11.7)	< 0.001
Acute Pancreatitis	31 (2.8)	12 (1.8)	19 (4.5)	2.7 (0.6 to 5.2)	0.009
Multiple transfusions	12 (1.1)	8 (1.2)	4 (0.9)	0.3 (− 1.3 to 1.6)	0.640
Others	33 (3.0)	20 (3.0)	13 (3.1)	0.1 (− 1.9 to 2.5)	0.925
Degree of ARDS severity, n (%)					
Severe	428 (38.8)	233 (34.4)	195 (45.9)	11.5 (5.6 to 17.4)	< 0.001
Moderate	674 (61.2)	444 (65.6)	230 (54.1)	11.5 (5.6 to 17.4)	< 0.001
APACHE II score, *mean* ± *SD*	21.0 ± 7.2 (**§**)	19.3 ± 6.6	23.6 ± 7.4	4.4 (3.5 to 5.1)	< 0.001
SOFA score, *mean* ± *SD*	9.3 ± 3.6	8.3 ± 3.1	10.9 ± 3.7	2.6 (2.2 to 3.0)	< 0.001
PaO_2_/FiO_2_, mmHg, *mean* ± *SD*	116.9 ± 39.6	120.9 ± 38.6	110.4 ± 40.4	10.5 (5.7 to 15.3)	< 0.001
FiO_2_*, mean* ± *SD*	0.78 ± 0.19	0.77 ± 0.19	0.80 ± 0.19	0.03 (0.01 to 0.05)	0.011
PaO_2_, mmHg, *mean* ± *SD*	86.8 ± 26.2	89.1 ± 26.9	83.1 ± 24.6	6.0 (2.8 to 9.2)	< 0.001
PaCO_2_, mmHg, *mean* ± *SD*	49.3 ± 12.9	48.1 ± 12.0	51.1 ± 14.1	3.0 (1.4 to 4.6)	< 0.001
pH, *mean* ± *SD*	7.30 ± 0.11	7.32 ± 0.10	7.27 ± 0.12	0.05 (0.04 to 0.06)	< 0.001
VT, mL/kg PBW, *mean* ± *SD*	6.8 ± 1.1	6.9 ± 1.0	6.8 ± 1.1	0.1 (− 0.03 to 0.23)	0.120
Respiratory rate, *mean* ± *SD*	21.5 ± 4.9	21.3 ± 4.6	21.8 ± 5.3	0.5 (− 0.1 to 1.1)	0.098
Minute ventilation, L/min, *mean* ± *SD*	9.2 ± 2.2	9.1 ± 2.1	9.2 ± 2.3	0.1 (− 0.2 to 0.4)	0.459
PEEP, cmH_2_O, *mean* ± *SD*	11.7 ± 3.3	11.7 ± 3.3	11.8 ± 3.2	0.1 (− 0.3 to 0.5)	0.620
Plateau pressure, *cmH*_*2*_*O, mean* ± *SD*	26.1 ± 4.9 (**¶**)	25.3 ± 4.8	27.4 ± 4.8	2.1 (1.5 to 2.7)	< 0.001
Driving pressure*, cmH*_*2*_*O, mean* ± *SD*	14.4 ± 4.8	13.7 ± 4.5	15.7 ± 5.1	2.0 (1.4 to 2.6)	< 0.001
No. extrapulmonary OF, mean ± SD	1.7 ± 1.1	1.4 ± 1.0	2.2 ± 1.2	0.8 (0.7 to 0.9)	< 0.001
Days on ventilatory support from diagnosis of moderate/severe ARDS, *mean* ± *SD*	11.1 ± 7.2	12.1 ± 6.8	9.4 ± 7.4	2.7 (1.8 to 3.6)	< 0.001
All-cause ICU mortality, n (%)	425 (38.6)	–	–	–	–
All-cause hospital mortality, n (%)	464 (42.1)	–	–	3.5 (− 0.6 to 7.6)	0.090

(*) Mean difference represents the difference between values in survivors and non-survivors.

APACHE, acute physiology and chronic health evaluation; ARDS, acute respiratory distress syndrome; CI, confidence interval; FiO_2_, fraction of inspired oxygen concentration; ICU, intensive care unit; OF, organ failure; PBW, predicted body weight; PEEP, positive end-expiratory pressure; SD, standard deviation; SOFA, sequential organ failure assessment scale; VT, tidal volume.

(§) APACHE II was missing in 10 survivors and 6 non-survivors.

(¶) Plateau pressure was missing in 4 survivors and 6 non-survivors.

**Figure 2:**
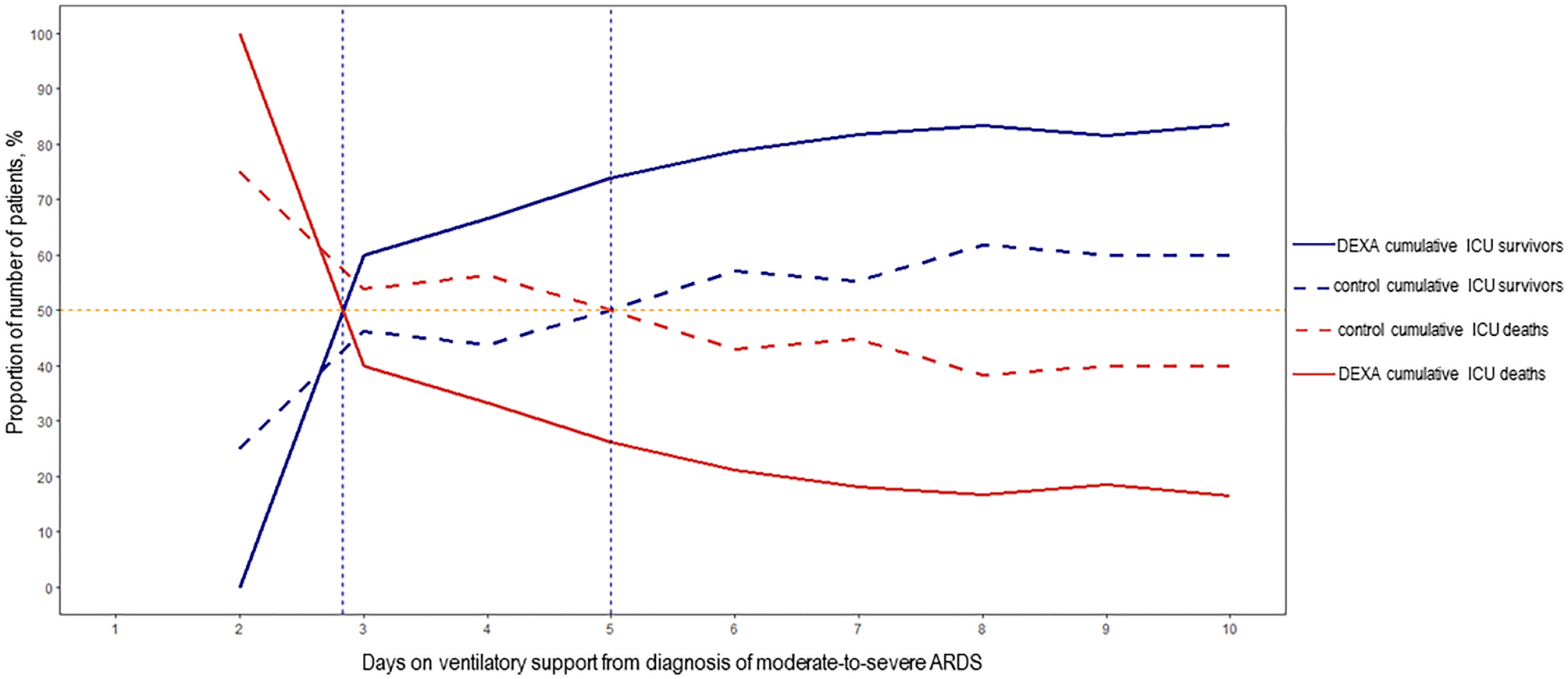
Percentage of cumulative number of survivors and non-survivors in the control group (n = 138) and in the dexamethasone arm (n = 139) in 277 patients with moderate-to-severe ARDS enrolled in a successful clinical trial. Each day represents the cumulative percentage of patients that have been mechanically ventilated up to that day and were discharged alive or dead at ICU discharge (see Supplementary Table S6 for details). *Note: for a better view of the marked difference between the crossing points of control and treatment arms, cumulative curves of ICU survivors and ICU deaths are represented within the first 10 days after diagnosis of moderate/severe ARDS. Abbreviations: DEXA represents the dexamethasone arm.*

Regarding the RCT, the cumulative proportion of all survivors and non-survivors in the DEXA-ARDS trial crossed at day-3 after meeting criteria for moderate/severe ARDS, 4 days earlier than in the observational cohort (Table S5). However, when considering both treatment arms separately, the proportion of survivors and non-survivors in the control group crossed at day-5 after meeting moderate/severe ARDS diagnosis, whereas in the dexamethasone group crossed before day-3 (Table S6, Fig. 2). The cumulative ICU mortality at day-7 was higher in the control group than in the treatment arm (13/29, 44.8% vs. 8/44, 18.2%; RR 2.5, 95% CI 1.2 to 5.2, *p* = 0.018). Collectively, the number of survivors in the trial cohort of 277 patients exceeded the number of non-survivors after day-3. A total of 73 patients were on MV for ≤ 7 days, and 21 patients died in ICU, representing a cumulative mortality of 28.8% (21/73) and an overall ICU mortality of 7.6% (21/277) (Table S5). Of note, in the DEXA-ARDS trial, there was an interim analysis for both efficacy and futility with blinded data from the first 157 enrolled patients with outcome data^14^. From the first 157 enrolled patients, 42 patients were ventilated for ≤ 7 days: 23 patients in the dexamethasone arm [3 ICU deaths (13.0%) and 20 ICU survivors (87.0%); 19 patients in the control arm [10 ICU deaths (52.6%) and 9 ICU survivors (47.4%)]. The mortality difference at 7-day was 39.6% (95% CI 11.3–61.4%; *p* = 0.006),

All-cause cumulative mortality at 28-days (63/233, 27.0%) was similar to overall ICU mortality (69/277, 24.9%; difference 2.1, 95% CI − 5.5 to 9.8, *p* = 0.585) with no relevant changes from day-28 to ICU discharge (Table S7).

## Discussion

The major findings of this study are: (i) ICU mortality for moderate-to-severe ARDS patients can be assessed using 28-day ICU mortality (according to our methodology); and (ii) assessment of ICU mortality within the first 7 days of study entry may be useful to inform and monitor effectiveness of interventions in clinical trials. We are unaware of studies reporting differences among crossing time-points between ICU survivors and non-survivors in observational cohorts versus RCTs.

Mortality is clearly an important outcome in clinical studies of ARDS given the high mortality^2,26,27^, and death in ARDS may be influenced by many modifiable and non-modifiable factors^14^. To date, no published studies have globally analyzed a comprehensive list of those factors in the specific context of ARDS. Some ARDS trials have reported survival benefits, including low VT^28^, high levels of PEEP^29^, prone positioning^30^, and dexamethasone^16^. However, marked heterogeneity existed with regard to the time-point of measurement and reporting mortality across these trials^17^. Consequently, little is known about the most appropriate or clinically relevant time-point to report or monitor mortality in clinical studies of ARDS. ICU mortality rate is meaningful to clinicians, patients and relatives^27^. However, it is plausible that mortality estimates at earlier time-points may predict outcomes and inform clinical decision-making. Additionally, early mortality estimates may provide useful information to clinicians and data safety monitoring committees.

Mortality is a crucial outcome that should be measured very precisely. Unfortunately, in many large observational and epidemiological studies, investigators have assumed that patients discharged from the hospital before day 28 were alive at day 28^31^. In our observational dataset of 1303 patients, we cannot provide the precise 28-day or 60-day mortality because 282 and 597 patients were discharged alive from the hospital before day 28 or day 60, respectively. Standardizing outcome measurements is the “sine qua non” of assessing improvement^32^. Inspired by previous studies of short-term and routinely reported outcomes in critically ill patients^28,33–38^ and controversies about meaningful outcomes and effect sizes^39^, we examined cumulative ICU mortality at different time-points in a large population of moderate/severe ARDS patients. We acknowledge that observational studies and RCTs in ARDS cannot be readily compared due to heterogeneity in outcomes reporting. There are some concerns related to the timing of enrollment into trials, since it is unclear whether patients are enrolled at the same point in their illness. In a systematic review of 67 RCTs of ARDS patients receiving lung-protective MV published between 2000 and 2019, a large unexplained variability was found in 28-day control group mortality, ranging from 10 to 67%^38^. Moreover, description of patient characteristics was often incomplete and commonly assessed ventilation variables were reported in a minority of trials. In our study, despite potential differences in patient population or in standards of care over a decade (2008–2018), ICU mortality rates were similar across all observational cohorts. We postulate that this may reflect the sequential inclusion of eligible patients, supporting the paradigm shift to pragmatic trials to enhance the generalizability of trial findings. A standardized time-point for measuring and reporting mortality that enables comparisons of lung-protective ventilation across trials of adjunctive therapies, pharmacologic and non-pharmacologic management strategies for ARDS^17^ may accelerate improvements in care and facilitate pooled and comparative analyses.

Death after a few days may not be a worse outcome than death after a few weeks. We found that assessment of 7-day outcome may be prognostically important for assessing the efficacy of a previously conducted RCT. A 7-day outcome may be useful as a proof of concept for interventions that improve very short-term mortality, as in patients with persistent ARDS, fulminant ARDS, or sub-phenotypes requiring rescue therapies^40^. This finding highlights one approach (retrospective analyses of prior datasets) to advance the field of ARDS research and improve short- and long-term outcome prediction. In this manner, we study patients to help predict what may happen to future similar patients treated in the same way^3^. In the era of pandemic medicine, when urgent therapeutic approaches are required quickly, it is plausible that the approach suggested in this study could identify therapeutic approaches earlier than using current endpoints. This progress will enable us to focus attention to precise therapies for most common causes of ARDS in combination with phenotyping work^41^. Consecutive assessment of mortality during the first week of MV after a standardized diagnosis of moderate-to-severe ARDS may be prognostically important and may inform trial implementation. By contrast, in six RCTs from the ARDS network, 10.5% of patients no longer met the ARDS criteria on the first day following enrollment, and increased over time^42^.

Our study has several strengths. First, since our observational data included consecutive ventilated patients with moderate/severe ARDS, we think that our findings represent real-life practice conditions, and results can be generalizable and applicable to everyday practice. Second, different to some studies in the field^42,43^, we did not have missing data on PaO_2_/FiO_2_ in the first day after diagnosis of moderate/severe ARDS, or missing data in the primary and secondary outcomes for the entire dataset of 1580 patients. Third, our findings support that 28-day ICU mortality approximates to overall ICU mortality. Fourth, 7-day ICU mortality may be clinically relevant when assessing RCTs testing therapeutic approaches in ARDS settings associated with early initial mortality.

Our study also has limitations. First, overall all-cause mortality is a crude measure that does not take in consideration the cause of death or the quality-of-life of ARDS survivors. In a previous report^12^, we found that most deaths in ARDS are not directly related to lung damage but to extrapulmonary OF. Second, although the observation that lines crossed before day-7 might mean that the tested treatment is beneficial, it was examined in a single RCT. Although the trial was not affected by treatment withdrawal, included patients with persistent moderate/severe ARDS at 24 h of initial diagnosis. However, we do not think that there is a relevant effect of time on our findings since the mean time between initiation of MV and diagnosis of moderate/severe ARDS was similar in the observational and trial cohorts. Also, most RCTs in ARDS conducted since 1990 considered enrolling patients within 24–48 h from ARDS diagnosis^44^. It is plausible that differences in 7-day mortality may be due to differences in the exclusion criteria of the trial^16^ and in the progress of supportive care in the last decade, but we think that dexamethasone was responsible for a reduction in mortality, as reported in our study and validated by the Recovery trial^35^.

## Conclusions

In summary, in our series of 1303 patients with moderate/severe ARDS included in the observational dataset, we have found that 28-day outcome (following our methodology) closely approximates and may be used as a surrogate for ICU mortality. In addition, we have identified a crossing point between cumulative survival and death in the ICU at 7 days. We also found that his intersection occurred before 7 days in a recently therapeutic successful clinical trial (as defined by a reduction of mortality) performed in patients with moderate-to-severe ARDS. Although this study focused on mortality, we acknowledge that the next frontier in ARDS clinical research will include longer-term outcomes, heterogeneity of treatment responses, and reflects the burden of health in survivors^45^.

## Supplementary Information

Supplementary Information.

## Data Availability

All data needed to evaluate the conclusions of this article are presented and tabulated in the main text or the Supplementary File. Data are available from the corresponding author on reasonable request.
