# Supplementary material for: Clinical relevance of timing of assessment of ICU mortality in patients with moderate-to-severe Acute Respiratory Distress Syndrome
Source: Sci Rep. 2023 Jan 27;13:1543. doi: 10.1038/s41598-023-28824-5 (PMC9883467; doi:10.1038/s41598-023-28824-5)
Supplement: Supplementary file 1 — Supplementary Information. [file 41598_2023_28824_MOESM1_ESM.docx]

**SUPPLEMENTARY FILE**

**Clinical relevance of timing of assessment of ICU outcome in patients with moderate-to-severe Acute Respiratory Distress Syndrome**

**Jesús Villar**, **Jesús M. González-Martin**, **José M. Añón**, **Carlos Ferrando**, **Juan A. Soler**, F**ernando Mosteiro**, **Juan M. Mora-Ordoñez**, **Alfonso Ambrós**, **Lorena Fernández**, **Raquel Montiel**, **Anxela Vidal**, **Tomás Muñoz**, **Lina Pérez-Méndez**, **Pedro Rodríguez-Suárez**, **Cristina Fernández**, **Rosa L. Fernández**, **Tamas Szakmany**, **Karen E.A. Burns**, **Ewout W. Steyerberg**, **Arthur S. Slutsky**

***Principal investigator and corresponding author:***

Jesús Villar

Multidisciplinary Organ Dysfunction Evaluation Research Network

Hospital Universitario Dr. Negrín

Barranco de la Ballena s/n, 4th Floor – South wing.

35019 Las Palmas de Gran Canaria, Spain.

Phone: +(34)606860027. Email: jesus.villar54@gmail.com

**SUPPLEMENTARY METHODS**

This ancillary analysis was an investigator-initiated study from prospective, multicenter clinical studies conducted in a network of intensive care units (ICUs) from several geographical areas of Spain.

**Ethics approval and consent to participate**

This study was approved by the Ethics Committee of Clinical Research at the Hospital Universitario Dr. Negrín (Las Palmas de Gran Canaria, Spain), and the requirement for informed consent was waived (Reference CEI/CEIm 2021-321-1) under the Royal Decree 1090/2015 of December 2015, and Royal Decree 957/2020 of November 2020 of the Spanish legislation for biomedical research based on the retrospective nature of the secondary analysis, the anonymization/dissociation of data, and with no harm and no benefit for the management of patients.

This study was conducted in accordance with the fundamental principles established in the Declaration of Helsinki**^1^**, the Convention of the European Council related to human rights and biomedicine, the Ethical Guidelines for Health-related Research Involving Humans by the Council for International Organization of Medical Sciences (CIOMS) of the World Health Organization (WHO), and within the requirements established by Spanish legislation for biomedical research, the protection of personal data, and bioethics. None of the findings reported in the present study have been published elsewhere. The study followed the STROBE (Strengthening the Reporting of Observational Studies in Epidemiology) guidelines for observational cohort studies**^2^**. This study is an extension of the Spanish Initiative for Epidemiology, Stratification and Therapies of ARDS (SIESTA) Program**^3-6^**.

**Patient population**

This is an ancillary analysis of an unrestricted set of data derived from 1580 adult patients with moderate-to-severe ARDS**^7^** treated with lung-protective ventilation in a network of ICUs under the Spanish Initiative for Epidemiology, Stratification, and Therapies of ARDS (SIESTA), as described previously (a full list of members and their affiliations appears at the end of this Supplementary Information). The study was conducted in two steps. In the first step, we analyzed 1303 patients included in four independent, multicenter, observational cohorts enrolling consecutive patients meeting the current criteria for moderate-to-severe ARDS**^7^**. In the ALIEN cohort**^3^** (NCT00736892. Registered 18 August 2008), 22 participating ICUs included 300 patients from September 2008 to May 2010, from which 255 patients were used to estimate the 1-year incidence of moderate/severe ARDS in 13 geographical areas of Spain. In the STANDARDS cohort (NCT02288949. Registered 13 November 2014), 24 participating ICUs included 300 patients from September 2013 to July 2015, and were used to quantify the risk of death in ARDS**^8^** and for testing whether driving pressure was superior to the variables that define it in predicting outcome in ARDS patients**^9^**. The STANDARDS-2 cohort was designed as a continuation of the STANDARDS cohort with the purpose of having a large database of ARDS patients combining our collective efforts. In the STANDARDS-2 cohort (NCT02836444. Registered 19 July 2016), 21 participating ICUs included 400 patients from August 2015 to April 2017, and were used to determine whether an enrichment strategy could be useful for selecting patients into future clinical trials**^10^**. In the PANDORA cohort (NCT03145974. Registered 9 May 2017), 303 consecutive patients with moderate-to-severe ARDS were included in a network of 22 ICUs from May 2017 to March 2018 (distributed in 3 periods of two consecutive months)**^6^**. From those patients, 301 patients were used as an external validation cohort for developing an ARDS score**^11^**.

In the second step, we tested the findings of our observational dataset on cumulative ICU survival from the time of moderate/severe ARDS diagnosis to ventilatory support discontinuation with a new cohort of 277 patients with moderate/severe ARDS enrolled in a successful (as defined by a reduction in mortality) therapeutic randomized clinical trial (DEXA-ARDS trial, ClinicalTrials.gov NCT01731795) testing dexamethasone in persistent moderate/severe ARDS**^12^**. We enrolled patients that would most likely respond to the intervention using a prognostic enrichment strategy to exclude patients with mild forms of ARDS after 24 hours of routine intensive care. With this new population from a successful RCT, we studied the relevance and external validity of measuring ICU mortality as primary endpoint in future clinical trials of ARDS.

**Study design**

Patients admitted to participating ICUs were screened daily during the study periods. All consecutive patients meeting the American-European Consensus Conference (AECC) criteria for ARDS**^13^** on positive end-expiratory pressure (PEEP) ≥5 cmH_2_O (in the ALIEN cohort) and the Berlin criteria for moderate or severe ARDS**^7^** (in the STANDARDS, STANDARDS-2, and PANDORA cohorts, and in the DEXA-ARDS trial) were included. By leaving the assessment of PaO_2_/FiO_2_ essentially unchanged, the AECC definition and the Berlin criteria are essentially identical**^7^**. The requirement of a minimum PEEP level of 5 cmH_2_O has no impact on the definition since patients with ARDS are managed with PEEP>5 cmH_2_O. Thus, our screening applies only to patients with moderate-to-severe ARDS, which include: (i) having an initiating clinical condition (pneumonia, aspiration, inhalation injury, overdose, sepsis, trauma, acute pancreatitis, etc.), (ii) within one week of a known clinical insult or new or worsening respiratory symptoms, (iii) bilateral pulmonary infiltrates on chest imaging (chest radiograph or computed tomography scan), (iv) absence of left atrial hypertension or no clinical signs of left heart failure, and (v) hypoxemia (as defined by a PaO_2_/FiO_2_ ≤100 mmHg on PEEP≥5 cmH_2_O for severe ARDS, and 100< PaO_2_/FiO_2_ ≤200 mmHg on PEEP≥5 cmH_2_O for moderate ARDS, regardless of FiO_2_). We did not enroll patients with persistent mild ARDS (PaO_2_/FiO_2_>200 mmHg during the entire ICU stay). However, we are confident that no patients with mild ARDS were excluded during our observational periods if they moved to a more severe category, although we do not have data on the precise number of those patients.

Day 0 (onset or diagnosis of moderate/severe ARDS) was defined as the day in which the patient first met moderate/severe ARDS criteria, irrespective of day of ICU admission or initiation of mechanical ventilation (MV). All patients had arterial blood gases at study inclusion. We did not use SpO_2_ as a surrogate for PaO_2_ for enrolling patients. For the purpose of this study and for appropriate identification of patients with moderate/severe ARDS, attending physicians considered only qualifying blood gases while patients were clinically stable, and did not consider transient falls in PaO_2_ resulting from acute events unrelated to the disease process (such as obstruction of endotracheal tube by secretions, endotracheal suctioning, ventilator disconnection, or sudden pneumothorax, among others). We excluded patients younger than 18 years old, patients with severe chronic pulmonary disease, acute cardiac failure, brain death, patients with a do-not-resuscitate orders, or postoperative patients receiving MV for <24 hours. Also, because diagnostic confusion could occur with other diseases and clinical situations that cause hypoxemia and have bilateral pulmonary infiltrates on radiographs, physicians excluded lymphangitic carcinoma, acute eosinophilic pneumonia, hypersensitivity pneumonitis, idiopathic pulmonary fibrosis, and others**^14^**.

**General care**

Attending clinicians followed current guidelines for general critical care management, which included the following: (i) in case of sepsis, physicians were urged to ensure early identification of causative microorganism, intravenous administration of antibiotics as soon as sepsis was suspected or recognized, and to optimize antibiotic selection and timely administration on the basis of antibiogram; (ii) fluid resuscitation and vasopressor use were individualized with the goal of maintaining a systolic blood pressure ≥90 mmHg or a mean arterial pressure ≥65 mmHg; (iii) to maintain hemoglobin between 7-10 g/dL.

For ventilatory management, clinicians followed current recommendations for lung-protective MV with a tidal volume (VT) of 4-8 ml/kg predicted body weight (PBW), a plateau pressure (Pplat) <30 cmH_2_O, a ventilatory rate (RR) to maintain a PaCO_2_ between 35-50 mmHg (permissive hypercapnia was allowed to target VT), and PEEP and FiO_2_ combinations according to the PEEP-FiO_2_ table of the ARDS protocol**^15^**, ensuring that among the PEEP and FiO_2_ combinations, clinicians should use the PEEP levels that allowed the reduction of FiO_2_ to the lowest level for maintaining a PaO_2_ within a target range of 60 to 100 mmHg or a peripheral capillary oxygen saturation (SpO_2_) within a target range of 90 to 98%.

The choice of drugs for sedation and analgesia, early neuromuscular blockade, prone positioning, recruitment maneuvers, hemodynamic management modalities, and the decision to perform a tracheotomy were left to the discretion of the attending physician. PBW was calculated using the following equations: 50 + 0.91 x [height (cm) – 152) for men and 45.5 + 0.91 x [height (cm) – 152] for women**^15^**. Although prone positioning and recruitment maneuvers were used in some patients, we do not have data on timing of prone positioning, or whether prone ventilation and recruitment maneuvers were applied as a rescue therapy, as a routine practice, or following any specific protocol.

Weaning off MV was not strictly protocolized, but could be started when the attending physician considered it clinically appropriate. Patients were assessed daily for readiness for a spontaneous breathing trial (SBT) based on the ARDSnet protocol**^15^**. In general, pre-requisites for the SBT included a partial reversal of the underlying cause of ARDS, a PaO_2_/FiO_2_>200 with PEEP<10 cmH_2_O and FiO_2_≤0.4, no vasopressors, continuous sedation minimized, and ability to cough during tracheal aspirations. Spontaneous ventilation was tested with a T-piece or with pressure support at 8 cmH_2_O. The duration of the SBT was at least 30 min and no longer than 120 minutes. If the patient passed the trial, a decision for extubation was taken, unless there was a specific reason not to extubate. Weaning and the decision to extubate were left to the discretion of the responsible clinician. Decisions about noninvasive ventilation, reintubation, or extubation were dictated by common clinical criteria.

**Data collection and follow-up**

Data were collected in each participating ICU using standardized case report forms, and transmitted to the coordinating center (Hospital Universitario Dr. Negrin) when the patient was discharged from hospital. Before exporting the data into a computerized database, a trained data collector from the coordinating center checked the completeness and the quality of information. Logical checks were performed for missing data and to find inconsistencies, especially regarding clinical diagnosis, date, and severity scores. If necessary, the data collector contacted the investigator by phone to validate the data or reformat the data for entry into the database.

**Outcomes**

Patients were followed-up until ICU and hospital discharge. We recorded date and status (alive or dead) of the patient at ICU and hospital discharge. Primary outcome was all-cause ICU mortality. For the purpose of our study, outcome at selected time-points were calculated considering the patient’s dependency on ventilatory support. For the 7-day outcome, we calculated the cumulative number of moderate/severe ARDS patients on ventilatory support for ≤7 days after diagnosis who were discharged alive or dead from the ICU, independently of the day in which patients were discharged from ICU. For the 28-day outcome, we calculated the cumulative number of patients on ventilatory support for ≤28 days who were discharged alive or dead from the ICU, independently of the day in which patients were discharged from ICU. For the 60-day outcome, we calculated the cumulative number of patients on ventilatory support for ≤60 days and discharged alive or dead from the ICU, independently of the day in which patients were discharged from ICU. No patients with extubation failure (need for reintubation or for continuing ventilatory support) were excluded from these calculations.

At each point-in-time, we analyzed differences in main etiologies, oxygenation defect, lung mechanics, and severity scores between survivors and non-survivors.

**Funding/Support**

This secondary analysis study was an academic, investigator-initiated, non-industry sponsored, non-interventional, multicenter, observational study. There was no specific funding source for this study.

The study coordinator and principal investigator (J. Villar) was funded by grants from the Instituto de Salud Carlos III, Madrid, Spain (PI16/00049, PI19/00141), by the European Reginal Development’s Funds, and the Asociación Científica Pulmón y Ventilación Mecánica (Las Palmas de Gran Canaria, Spain). J. Villar, J.M. Añón, and C. Ferrando received grant support from the Institute de Salud Carlos III, Madrid, Spain (CB06/06/1088). P. Rodríguez-Suárez was funded by the Health Service of the Autonomic Government of the Canary Islands, and by the Royal College of Physicians of Las Palmas de Gran Canaria, Spain. Funds were exclusively allocated for covering salary support of project data managers. A.S. Slutsky was funded by the Canadian Institutes of Health (grants #137772 and FDN 143285). None of the funding agencies supporting the principal investigators participated in the study design, data collection, analysis and interpretation of data, or in the writing and submission of the study report. All researchers are independent of their funding bodies. The authors would like to thank Mr. Yasser and Lily B of LP Bahrain for their support in loving memory of Lily Bendahan. Centers enrolling patients were the legal sponsors of the study in their own hospitals.

**Declaration of interests**

Authors declare no competing interests directly or indirectly related to this study. None of the clinical investigators received any honorarium for participating in the study.

**Coordination, conduct, and monitoring of the study**

Since a key lesson in the diagnostic process is synchronous communication (when all local investigators are present at the same time), real-time synchronous exchange of ideas and information, and interdisciplinary meetings to derive plans are invaluable**^16^**. Before enrolling the first patient in any of the four cohorts of the observational cohorts and in the RCT used in this study, the principal investigator, the data manager, and all site investigators from participating ICUs attended at least one formal session in Madrid, Spain, for discussing the study protocols and data collection on the standardized case report form (CRF). All documents required for each cohort, including the study protocol, management guidelines, copies of CRFs, model of an informed consent form (in case it was needed) were available for each attending investigator at each participating ICUs, for ensuring compliance with the daily patient screening and inclusion, with the study protocol, and with the data collection process.

Face-to-face meetings were held as determined by need. Routine research meetings were conducted in Madrid, Spain, or by email, fax, or teleconferencing. Since there are well documented issues of clinician‘s ability to recognize ARDS**^17^**, we monitored through discussion in regular meetings and by contacting site investigators after each study period. In order to avoid confirmation bias, we opted not to use the raw data in the CRF for ARDS identification. An electronic Newsletter for every cohort and for the trial regularly informed investigators on study conduct and or any relevant information. In every cohort, the coordinating center (Research Unit, Hospital Universitario Dr. Negrin, Las Palmas de Gran Canaria, Spain) took responsibility for: (i) communicating to participating ICUs, (ii) monitoring and supervising the progress of the cohort, (iii) informing and advising on all aspects of the study.

**SUPPLEMENTARY RESULTS**

**TABLE S1. Cumulative number of patients in the observational pooled cohort of 1303 patients with moderate-to-severe ARDS in relation to number of days on ventilatory support and status (survivor/dead) at ICU discharge.** Highlighted rows in yellow represent selected ICU outcomes in this study (7-day, 28-day, 60-day, ICU discharge). The annotation “>60” represents ICU discharge.

| Days on ventilatory support | Cumulative No. patients | Cumulative ICU survivor | Cumulative ICU deaths | Proportion survivors | Proportion deaths | Difference of proportions  95% CI |
| --- | --- | --- | --- | --- | --- | --- |
| 1 | 58 | 4 | 54 | 6.9 | 93.1 | -86.2 (-97.2 to -75.3) |
| 2 | 131 | 27 | 104 | 20.6 | 79.4 | -58.8 (-69.3 to -48.2) |
| 3 | 169 | 40 | 129 | 23.7 | 76.3 | -52.7 (-62.3 to -43.0) |
| 4 | 227 | 76 | 151 | 33.5 | 66.5 | -33.0 (-42.2 to -23.9) |
| 5 | 287 | 117 | 170 | 40.8 | 59.2 | -18.5 (-26.9 to -10.1) |
| 6 | 350 | 164 | 186 | 46.9 | 53.1 | -6.3 (-14.0 to 1.4) |
| 7 | 412 | 206 | 206 | 50 | 50 | 0 (-6.8 to 6.8) |
| 8 | 479 | 255 | 224 | 53.2 | 46.8 | 6.5 (-0.1 to 13.0) |
| 9 | 545 | 298 | 247 | 54.7 | 45.3 | 9.4 (3.3 to 15.5) |
| 10 | 585 | 332 | 253 | 56.8 | 43.3 | 13.5 (7.7 to 19.4) |
| 11 | 633 | 364 | 269 | 57.5 | 42.5 | 15.0 (9.4 to 20.6) |
| 12 | 680 | 394 | 286 | 57.9 | 42.1 | 15.9 (10.5 to 21.3) |
| 13 | 723 | 425 | 298 | 58.8 | 41.2 | 17.6 (12.4 to 22.8) |
| 14 | 773 | 455 | 318 | 58.9 | 41.1 | 17.7 (12.7 to 22.8) |
| 15 | 816 | 478 | 338 | 58.6 | 41.4 | 17.2 (12.3 to 22.1) |
| 16 | 851 | 503 | 348 | 59.1 | 40.9 | 18.2 (13.4 to 23.0) |
| 17 | 879 | 525 | 354 | 59.7 | 40.3 | 19.5 (14.8 to 24.2) |
| 18 | 913 | 550 | 363 | 60.2 | 39.8 | 20.5 (15.9 to 25.1) |
| 19 | 936 | 564 | 372 | 60.3 | 39.7 | 20.5 (16.0 to 25.1) |
| 20 | 955 | 575 | 380 | 60.2 | 39.8 | 20.4 (15.9 to 24.9) |
| 21 | 983 | 594 | 389 | 60.4 | 39.6 | 20.9 (16.4 to 25.3) |
| 22 | 1003 | 607 | 396 | 60.5 | 39.5 | 21.0 (16.7 to 25.4) |
| 23 | 1023 | 622 | 401 | 60.8 | 39.2 | 21.6 (17.3 to 25.9) |
| 24 | 1041 | 634 | 407 | 60.9 | 39.1 | 21.8 (17.5 to 26.1) |
| 25 | 1060 | 648 | 412 | 61.1 | 38.9 | 22.3 (18.0 to 26.5) |
| 26 | 1073 | 655 | 418 | 61.0 | 39.0 | 22.1 (17.9 to 26.3) |
| 27 | 1087 | 664 | 423 | 61.1 | 38.9 | 22.2 (18.0 to 26.4) |
| 28 | 1102 | 677 | 425 | 61.4 | 38.6 | 22.9 (18.7 to 27.0) |
| 29 | 1114 | 685 | 429 | 61.5 | 38.5 | 23.0 (18.9 to 27.1) |
| 30 | 1130 | 695 | 435 | 61.5 | 38.5 | 23.0 (18.9 to 27.1) |
| 31 | 1138 | 698 | 440 | 61.3 | 38.7 | 22.7 (18.6 to 26.8) |
| 32 | 1145 | 703 | 442 | 61.4 | 38.6 | 22.8 (18.7 to 26.9) |
| 33 | 1159 | 714 | 445 | 61.6 | 38.4 | 23.2 (19.2 to 27.3) |
| 34 | 1167 | 720 | 447 | 61.7 | 38.3 | 23.4 (19.4 to 27.4) |
| 35 | 1173 | 724 | 449 | 61.7 | 38.3 | 23.4 (19.4 to 27.5) |
| 36 | 1178 | 729 | 449 | 61.9 | 38.1 | 23.8 (19.8 to 27.8) |
| 37 | 1180 | 730 | 450 | 61.9 | 38.1 | 23.7 (19.7 to 27.7) |
| 38 | 1186 | 733 | 453 | 61.8 | 38.2 | 23.6 (19.6 to 27.6) |
| 39 | 1188 | 735 | 453 | 61.9 | 38.1 | 23.7 (19.8 to 27.7) |
| 40 | 1192 | 738 | 454 | 61.9 | 38.1 | 23.8 (19.8 to 27.8) |
| 41 | 1201 | 746 | 455 | 62.1 | 37.9 | 24.2 (20.3 to 28.2) |
| 42 | 1205 | 749 | 456 | 62.2 | 37.8 | 24.3 (20.4 to 28.3) |
| 43 | 1213 | 757 | 456 | 62.4 | 37.6 | 24.8 (20.9 to 28.8) |
| 44 | 1217 | 760 | 457 | 62.5 | 37.6 | 24.9 (21.0 to 28.8) |
| 45 | 1221 | 763 | 458 | 62.5 | 37.5 | 25.0 (21.1 to 28.9) |
| 46 | 1226 | 764 | 462 | 62.3 | 37.7 | 24.6 (20.7 to 28.6) |
| 47 | 1226 | 764 | 462 | 62.3 | 37.7 | 24.6 (20.7 to 28.6) |
| 48 | 1235 | 769 | 466 | 62.3 | 37.7 | 24.5 (20.6 to 28.4) |
| 49 | 1237 | 771 | 466 | 62.3 | 37.7 | 24.7 (20.8 to 28.6) |
| 50 | 1242 | 774 | 468 | 62.3 | 37.7 | 24.6 (20.8 to 28.5) |
| 51 | 1246 | 777 | 469 | 62.4 | 37.6 | 24.7 (20.8 to 28.6) |
| 52 | 1250 | 780 | 470 | 62.4 | 37.6 | 24.8 (20.9 to 28.7) |
| 53 | 1256 | 786 | 470 | 62.6 | 37.4 | 25.2 (21.3 to 29.0) |
| 54 | 1257 | 786 | 471 | 62.5 | 37.5 | 25.1 (21.2 to 28.9) |
| 55 | 1260 | 788 | 472 | 62.5 | 37.5 | 25.1 (21.2 to 28.9) |
| 56 | 1262 | 789 | 473 | 62.5 | 37.5 | 25.0 (21.2 to 28.9) |
| 57 | 1264 | 790 | 474 | 62.5 | 37.5 | 25.0 (21.2 to 28.9) |
| 58 | 1265 | 790 | 475 | 62.5 | 37.6 | 24.9 (21.1 to 28.8) |
| 59 | 1267 | 792 | 475 | 62.5 | 37.5 | 25.0 (21.2 to 28.9) |
| 60 | 1269 | 794 | 475 | 62.6 | 37.4 | 25.1 (21.3 to 29.0) |
| >60 | 1303 | 816 | 487 | 62.6 | 37.4 | 25.3 (21.5 to 29.0) |

*ARDS: acute respiratory distress syndrome; CI: confidence interval; ICU: intensive care unit; No.: number.*

**TABLE S2. Baseline characteristics and outcome of 1,269 patients with moderate-to-severe acute respiratory distress syndrome.** Number of patients represents the cumulative number of patients on ventilatory support for ≤60 days who were discharged dead or alive from the ICU, independently of the day in which patients were discharged from ICU.

| **Variables** | **All patients**  **N= 1269** | **survivors**  **N= 794** | **Non-survivors**  **N= 475** | **Mean difference**  **(95% CI)** | **p-value** |
| --- | --- | --- | --- | --- | --- |
| Age, *years, mean±SD* | 57.1±15.8 | 54.1±15.8 | 62.0±14.6 | 7.9 (6.2 to 9.6) | <0.001 |
| Sex, *n (%)*  Male  Female | 877 (69.1)  392 (30.9) | 550 (69.3)  244 (30.7) | 327 (68.8)  148 (31.2) | 0.5 (-4.7 to 5.8) | 0.852 |
| Etiology, *n (%)*  Pneumonia  Sepsis  Aspiration  Trauma  Acute Pancreatitis  Multiple transfusions  Others | 566 (44.6)  358 (28.2)  140 (11.0)  111 (8.8)  44 (3.5)  13 (1.0)  37 (2.9) | 363 (45.7)  198 (24.9)  89 (11.2)  95 (12.0)  18 (2.3)  9 (1.1)  22 (2.8) | 203 (42.7)  160 (33.7)  51 (10.7)  16 (3.4)  26 (5.5)  4 (0.8)  15 (3.2) | 3.0 (-2.7 to 8.6)  8.8 (3.6 to 14.0)  0.5 (-3.2 to 3.9)  8.6 (5.7 to 11.4)  3.2 (1.1 to 5.8)  0.3 (-1.1 to 1.4)  0.4 (-1.5 to 2.6) | 0.298  <0.001  0.783  <0.001  0.003  0.601  0.684 |
| Degree of ARDS severity, *n (%)*  Severe  Moderate | 498 (39.2)  771 (60.8) | 282 (35.5)  512 (64.5) | 216 (45.5)  259 (54.5) | 10.0 (4.4 to 15.5) | <0.001 |
| APACHE II score, *mean±SD* | 21.0±7.0 (**§**) | 19.5±6.6 | 23.3±7.1 | 3.8 (3.0 to 4.6) | <0.001 |
| SOFA score, *mean±SD* | 9.3±3.5 | 8.5±3.1 | 10.7±3.7 | 2.2 (1.8 to 2.6) | <0.001 |
| PaO_2_/FiO_2_, *mmHg, mean±SD* | 116.1±39.2 | 119.3±38.4 | 110.9±40.0 | 8.4 (4.0 to 12.8) | <0.001 |
| FiO_2_, *mean±SD* | 0.78±0.19 | 0.78±0.19 | 0.80±0.19 | 0.02 (-0.02 - 0.04) | 0.070 |
| PaO_2_, *mmHg, mean±SD* | 86.2±26.2 | 88.2±26.7 | 83.0±25.0 | 5.2 (2.2 to 8.2) | <0.001 |
| PaCO_2_, *mmHg, mean±SD* | 49.4±12.9 | 48.5±12.3 | 50.8±13.8 | 2.3 (0.8 to 3.8) | 0.002 |
| pH, *mean±SD* | 7.30±0.11 | 7.31±0.10 | 7.28±0.12 | 0.03 (0.02 to 0.04) | <0.001 |
| VT, *mL/kg PBW, mean±SD* | 6.8±1.1 | 6.9±1.0 | 6.8±1.1 | 0.1 (-0.02 to 0.2) | 0.097 |
| Respiratory rate, *mean±SD* | 21.5±4.9 | 21.4±4.6 | 21.8±5.2 | 0.4 (-0.2 to 1.0) | 0.154 |
| Minute ventilation, *L/min, mean±SD* | 9.2±2.2 | 9.2±2.1 | 9.1±2.3 | 0.1 (-0.2 to 0.4) | 0.429 |
| PEEP, *cmH_2_O, mean±SD* | 11.8±3.3 | 11.8±3.3 | 11.7±3.2 | 0.1 (-0.3 to 0.5) | 0.597 |
| Plateau pressure, *cmH_2_O, mean±SD* | 26.2±4.9 (**¶**) | 25.5±4.8 | 27.2±4.9 | 1.7 (1.2 to 2.3) | <0.001 |
| Driving pressure, *cmH_2_O, mean±SD* | 14.4±4.8 | 13.8±4.5 | 15.6±5.1 | 1.8 (1.3 to 2.3) | <0.001 |
| No. extrapulmonary OF, *mean±SD* | 1.7±1.1 | 1.5±1.0 | 2.1±1.2 | 0.6 (0.5 to 0.7) | <0.001 |
| Days on ventilatory support from moderate/severe ARDS diagnosis,  *d, mean±SD* | 14.9±12.3 | 16.2±12.3 | 12.6±11.9 | 3.6 (2.2 to 5.0) | <0.001 |
| All-cause ICU mortality, *n (%)* | 475 (37.4) | - | - | - | - |
| All-cause hospital mortality, *n (%)* | 524 (41.3) | - | - | 3.9 (-1.6 to 9.5) | 0.168 |

*APACHE: acute physiology and chronic health evaluation; ARDS: acute respiratory distress syndrome; CI: confidence interval; d: days; FiO_2_: fraction of inspired oxygen concentration; ICU: intensive care unit; OF: organ failure; PBW: predicted body weight; PEEP: positive end-expiratory pressure; SD: standard deviation; SOFA: sequential organ failure assessment scale; VT: tidal volume.*

*(§) APACHE II was missing in 11 survivors and 8 non-survivors.*

*(¶) Plateau pressure was missing in 6 survivors and 9 non-survivors.*

**TABLE S3. Cumulative mortality of 1,303 patients with moderate-to severe ARDS within each pooled cohort at day-7, day-28, day 60 in the ICU after diagnosis of moderate/severe ARDS, and at ICU discharge.**

| **Outcomes** | **Cohorts** | | | |  |
| --- | --- | --- | --- | --- | --- |
|  | ALIEN | STANDARDS | STANDARDS-2 | PANDORA | **p-value** |
| **Ventilatory support ≤7 days**  No. patients  No. Deaths  Death rate (95%CI)  Risk ratio (95%CI) | 85  47  55.3 (44.7-65.9)  1 (Ref) | 85  45  52.9 (42.3-63.6)  0.96 (0.73-1.26) | 113  51  45.1 (36.0-54.3)  0.82 (0.62-1.08) | 129  63  48.8 (40.2-57.5)  0.88 (0.68-1.15) | 0.496 |
| **Ventilatory support≤28 days**  No. patients  No. Deaths  Death rate (95%CI)  Risk ratio (95%CI) | 247  105  42.5 (36.4-48.7)  1 (Ref) | 256  103  40.2 (34.2-46.2)  0.95 (0.77-1.17) | 321  112  34.9 (29.7-40.1)  0.82 (0.67-1.01) | 278  105  37.8 (32.1-43.5)  0.89 (0.72-1.10) | 0.280 |
| **Ventilatory support≤60 days**  No. patients  No. Deaths  Death rate (95%CI)  Risk ratio (95%CI) | 291  119  40.9 (35.2-46.5)  1 (Ref) | 293  111  37.9 (32.3-43.4)  0.93 (0.76-1.13) | 386  133  34.5 (29.7-39.2)  0.84 (0.69-1.02) | 299  112  37.5 (32.0-42.9)  0.92 (0.75-1.12) | 0.396 |
| **ICU discharge**  No. patients  No. Deaths  Death rate (95%CI)  Risk ratio (95%CI) | 300  123  41.0 (35.5-46.6)  1 (Ref) | 300  114  38.0 (32.5-43.5)  0.93 (0.76-1.13) | 400  138  34.5 (29.8-39.2)  0.84 (0.69-1.02) | 303  112  37.0 (31.5-42.4)  0.90 (0.74-1.10) | 0.366 |

**TABLE S4. Characteristics and outcome data of 1298 patients assessed under a standardized ventilatory approach at 24 h after diagnosis of moderate-to-severe acute respiratory distress syndrome (ARDS).** Five out of 1303 patients died in the first few hours after been enrolled.

| **Variables** | **All patients**  **N=1,298** | **Survivors**  **N= 816** | **Non-survivors**  **N= 482** | **Mean difference**  **(95%CI)** | **p-value** |
| --- | --- | --- | --- | --- | --- |
| Age, *years, mean±SD* | 57.2±15.7 | 54.3±15.7 | 62.1±14.5 | 7.8 (6.1 to 9.5) | <0.001 |
| Sex, *n (%)*  Male  Female | 900 (69.3)  398 (30.7) | 566 (69.4)  250 (30.6) | 334 (69.3)  148 (30.7) | 0.1 (-5.0 to 5.3) | 0.970 |
| Etiology*, n (%)*  Pneumonia  Sepsis  Aspiration  Trauma  Acute Pancreatitis  Multiple transfusions  Others | 587 (45.2)  363 (28.0)  140 (10.8)  112 (8.6)  45 (3.5)  13 (1.0)  38 (2.9) | 378 (46.3)  201 (24.6)  90 (11.0)  96 (11.8)  19 (2.3)  9 (1.1)  23 (2.8) | 209 (43.4)  162 (33.6)  50 (10.4)  16 (3.3)  26 (5.4)  4 (0.8)  15 (3.1) | 2.9 (-2.7 to 8.4)  9.0 (3.9 to 14.2)  0.6 (-3.0 to 4.0)  8.5 (5.6 to 11.2)  3.1 (1.0 to 5.6)  0.3 (-1.1 to 1.4)  0.3 (-1.5 to 2.5) | 0.311  <0.001  0.736  <0.001  0.003  0.598  0.756 |
| Degree of ARDS severity, *n (%)*  Severe  Moderate  Mild  PaO_2_/FiO_2_>300 | 268 (20.6)  775 (59.7)  223 (17.2)  32 (2.5) | 90 (11.0)  515 (63.1)  181 (22.2)  30 (3.7) | 178 (36.9)  260 (53.9)  42 (8.7)  2 (0.4) | 25.9 (21.1 to 30.7)  9.2 (3.7 to 14.7)  13.5 (9.6 to 17.2)  3.3 (1.8 to 4.9) | <0.001  0.001  <0.001  <0.001 |
| APACHE II score, *mean±SD* | 19.2±7.4 (**§**) | 17.3±6.8 | 22.4±7.3 | 5.1 (4.3 to 5.9) | <0.001 |
| SOFA score, *mean±SD* | 9.2±3.8 ♣ | 8.2±3.3 | 11.1±3.9 | 2.9 (2.5 to 3.3) | <0.001 |
| PaO_2_/FiO_2_, *mmHg,* *mean±SD* | 152.6±61.8 | 168.0±61.9 | 126.5±52.2 | 41.5 (34.9 to 48.1) | <0.001 |
| FiO_2_, *mean±SD* | 0.65±0.18 | 0.61±0.16 | 0.72±0.19 | 0.11 (0.09 to 0.13) | <0.001 |
| PaO_2_, *mmHg, mean±SD* | 92.6±28.3 | 97.4±29.2 | 84.4±24.7 | 13.0 (9.9 to 16.1) | <0.001 |
| PaCO_2_, *mmHg, mean±SD* | 46.9±10.3 | 45.5±9.1 | 49.4±11.7 | 3.9 (2.8 to 5.0) | <0.001 |
| pH, *mean±SD* | 7.34±0.09 | 7.36±0.08 | 7.30±0.11 | 0.06 (0.04 to 0.07) | <0.001 |
| VT, *mL/kg PBW, mean±SD* | 6.7 ±1.0 | 6.8±0.9 | 6.6±1.0 | 0.2 (0.1 to 0.3) | <0.001 |
| Respiratory rate, *mean±SD* | 23.4±5.0 | 22.8±4.9 | 24.3±5.2 | 1.5 (0.9 to 2.1) | <0.001 |
| Minute ventilation, *L/min, mean±SD* | 9.8 ±2.3 | 9.7±2.2 | 9.9 ±2.3 | 0.2 (-0.05 to 0.45) | 0.120 |
| PEEP, *cmH_2_O, mean±SD* | 12.3±3.0 | 12.2±3.0 | 12.5±3.0 | 0.3 (0.004 to 0.64) | 0.082 |
| Plateau pressure, *cmH_2_O, mean±SD* | 26.3±4.7 | 24.9±4.2 | 28.8±4.5 | 3.9 (3.4 to 4.4) | <0.001 |
| Driving pressure, *cmH_2_O, mean±SD* | 14.1±4.5 | 12.8±3.9 | 16.3±4.7 | 3.5 (3.0 to 4.0) | <0.001 |
| No. extrapulmonary OF, *mean±SD* | 1.8±1.2 | 1.5±1.0 | 2.4±1.2 | 0.9 (0.8 to 1.0) | <0.001 |
| Ventilatory support from diagnosis of moderate/severe ARDS, *d, mean±SD* | 16.7±17.0 | 18.1±17.2 | 14.4±16.4 | 3.7 (1.8 to 5.6) | <0.001 |
| All-cause ICU mortality, *n (%)* | 482 (37.1) | - | - | - | - |
| All-cause hospital mortality, *n (%)* | 534 (41.1) | - | - | 4.0 (0.2 to 7.7) | 0.037 |

*APACHE: acute physiology and chronic health evaluation; ARDS: acute respiratory distress syndrome; CI: confidence interval; d: days; FiO_2_: fraction of inspired oxygen concentration; ICU: intensive care unit; OF: organ failure; PBW: predicted body weight; PEEP: positive end-expiratory pressure; SD: standard deviation; SOFA: sequential organ failure assessment scale; VT: tidal volume.*

*(§) APACHE II was missing in 77 survivors and 57 non-survivors.*

*(♣) SOFA score was missing in 3 survivors and 2 non-survivors.*

**TABLE S5. Cumulative number of patients in the “trial cohort” of 277 patients with moderate-to-severe ARDS in relation to the number of days on ventilatory support and status (survivor/dead) at ICU discharge.** Cumulative proportion of survivors and non-survivors for the entire trial crossed at day-3 after diagnosis of moderate/severe ARDS. Highlighted rows in yellow represent selected ICU outcomes in this study (7-day, 28-day, 60-day, ICU discharge). The annotation “>60” represents ICU discharge.

| **Days on ventilatory support** | **Cumulative No. patients** | **Cumulative ICU survivors** | **Cumulative ICU deaths** | **Proportion survivors** | **Proportion deaths** | **Difference of proportions**  **95% CI** |
| --- | --- | --- | --- | --- | --- | --- |
| 1 | 0 | 0 | 0 | 0 | 0 | - |
| 2 | 6 | 1 | 5 | 16.7 | 83.3 | -66.8 (-100 to -7.8) |
| 3 | 18 | 9 | 9 | 50.0 | 50.0 | 0 (-32.7 to 32.7 |
| 4 | 28 | 15 | 13 | 53.6 | 46.4 | 7.1 (-22.6 to 36.8) |
| 5 | 43 | 27 | 16 | 62.8 | 37.2 | 25.6 (2.8 to 48.3) |
| 6 | 61 | 42 | 19 | 68.9 | 31.2 | 37.7 (19.6 to 55.8) |
| 7 | 73 | 52 | 21 | 71.2 | 28.8 | 42.5 (26.4 to 58.5) |
| 8 | 88 | 66 | 22 | 75.0 | 25.0 | 50.0 (36.1 to 63.9) |
| 9 | 100 | 74 | 26 | 74.0 | 26.0 | 48.0 (34.8 to 61.2) |
| 10 | 113 | 85 | 28 | 75.2 | 24.8 | 50.4 (38.3 to 62.6) |
| 11 | 123 | 91 | 32 | 74.0 | 26,0 | 48.0 (36.2 to 59.8) |
| 12 | 135 | 98 | 37 | 72.6 | 27.4 | 45.2 (33.8 to 56.6) |
| 13 | 149 | 110 | 39 | 73.8 | 26.2 | 47.6 (37.0 to 58.3) |
| 14 | 156 | 115 | 41 | 73.7 | 26.3 | 47.4 (37.0 to 57.9) |
| 15 | 167 | 120 | 47 | 71.9 | 28,1 | 43.7 (33.5 to 54.0) |
| 16 | 180 | 131 | 49 | 72.8 | 27.2 | 45.6 (35.8 to 55.3) |
| 17 | 186 | 134 | 52 | 72.0 | 28.0 | 44.1 (34.4 to 53.7) |
| 18 | 192 | 140 | 52 | 72,9 | 27.1 | 45.8 (36.4 to 55.2) |
| 19 | 195 | 143 | 52 | 73.3 | 26.7 | 46.7 (37.4 to 56.0) |
| 20 | 199 | 146 | 53 | 73.4 | 26.6 | 46.7 (37.6 to 55.9) |
| 21 | 204 | 148 | 56 | 72.5 | 27.5 | 45.1 (36.0 to 54.3) |
| 22 | 209 | 150 | 59 | 71.8 | 28.2 | 43.5 (34.4 to 52.7) |
| 23 | 212 | 153 | 59 | 72.2 | 27.8 | 44.3 (35.3 to 53.3) |
| 24 | 214 | 154 | 60 | 72.0 | 28.0 | 43.9 (35.0 to 52.9) |
| 25 | 217 | 156 | 61 | 71.9 | 28.1 | 43.8 (34.9 to 52.7) |
| 26 | 222 | 160 | 62 | 72.1 | 27.9 | 44.1 (35.4 to 52.9) |
| 27 | 230 | 167 | 63 | 72.6 | 27.4 | 45.2 (36.6 to 53.8) |
| 28 | 233 | 170 | 63 | 73.0 | 27.0 | 45.9 (37.4 to 54.4) |
| 29 | 234 | 170 | 64 | 72.7 | 27.3 | 45.3 (36.8 to 53.8) |
| 30 | 238 | 174 | 64 | 73.1 | 26.9 | 46.2 (37.8 to 54.6) |
| 31 | 239 | 175 | 64 | 73.2 | 26.8 | 46.4 (38.1 to 54.8) |
| 32 | 241 | 177 | 64 | 73.4 | 26.6 | 46.9 (38.6 to 55.2) |
| 33 | 248 | 183 | 65 | 73.8 | 26.2 | 47.6 (39.4 to 55.7) |
| 34 | 249 | 184 | 65 | 73.9 | 26.1 | 47.8 (39.7 to 55.9) |
| 35 | 251 | 186 | 65 | 74.1 | 25.9 | 48.2 (40.1 to 56.3) |
| 36 | 253 | 188 | 65 | 74.3 | 25.7 | 48.6 (40.6 to 56.6) |
| 37 | 253 | 188 | 65 | 74.3 | 25.7 | 48.6 (40.6 to 56.6) |
| 38 | 255 | 189 | 66 | 74.1 | 25,9 | 48.2 (40.2 to 56.2) |
| 39 | 255 | 189 | 66 | 74.1 | 25.9 | 48.2 (40.2 to 56.2) |
| 40 | 257 | 191 | 66 | 74.3 | 25.7 | 48.6 (40.7 to 56.6) |
| 41 | 259 | 193 | 66 | 74.5 | 25.5 | 49.0 (41.1 to 56.9) |
| 42 | 261 | 195 | 66 | 74.7 | 25.3 | 49.4 (41.6 to 57.3) |
| 43 | 262 | 196 | 66 | 74.8 | 25.2 | 49.6 (41.8 to 57.4) |
| 44 | 262 | 196 | 66 | 74.8 | 25.2 | 49.6 (41.8 to 57.4) |
| 45 | 264 | 198 | 66 | 75.0 | 25.0 | 50.0 (42.2 to 57.8) |
| 46 | 264 | 198 | 66 | 75.0 | 25.0 | 50.0 (42.2 to 57.8) |
| 47 | 264 | 198 | 66 | 75.0 | 25.0 | 50.0 (42.2 to 57.8) |
| 48 | 266 | 199 | 67 | 74.8 | 25.2 | 49.6 (41.9 to 57.4) |
| 49 | 266 | 199 | 67 | 74.8 | 25.2 | 49.6 (41.9 to 57.4) |
| 50 | 267 | 200 | 67 | 74.9 | 25.1 | 49.8 (42.1 to 57.5) |
| 51 | 268 | 200 | 68 | 74.6 | 25.4 | 49.2 (41.5 to 57.0) |
| 52 | 269 | 201 | 68 | 74.7 | 25.3 | 49.4 (41.7 to 57.2) |
| 53 | 272 | 204 | 68 | 75.0 | 25.0 | 50.0 (42.4 to 57.7) |
| 54 | 272 | 204 | 68 | 75.0 | 25.0 | 50.0 (42.4 to 57.7) |
| 55 | 272 | 204 | 68 | 75.0 | 25.0 | 50.0 (42.4 to 57.7) |
| 56 | 272 | 204 | 68 | 75.0 | 25.0 | 50.0 (42.4 to 57.7) |
| 57 | 272 | 204 | 68 | 75.0 | 25.0 | 50.0 (42.4 to 57.7) |
| 58 | 272 | 204 | 68 | 75.0 | 25.0 | 50.0 (42.4 to 57.7) |
| 59 | 273 | 205 | 68 | 75.1 | 24.9 | 50.2 (42.6 to 57.8) |
| 60 | 274 | 206 | 68 | 75.2 | 24.8 | 50.4 (42.8 to 58.0) |
| >60 | 277 | 208 | 69 | 75.1 | 24.9 | 50.2 (42.6 to 57.7) |

*ARDS: acute respiratory distress syndrome; CI: confidence interval; ICU: intensive care unit; No.: number.*

**TABLE S6. Cumulative number of patients in the “trial cohort” by treatment groups of 277 patients with moderate-to-severe ARDS in relation to the number of days on ventilatory support and status (survivor/dead) at ICU discharge.** Highlighted rows in yellow represent selected ICU outcomes. The annotation “>60” represents at ICU discharge.

| **Days on ventilatory support** | **Control group (n=138)** | | | | | | **Dexamethasone group (n=139)** | | | | | |
| --- | --- | --- | --- | --- | --- | --- | --- | --- | --- | --- | --- | --- |
|  | Cumulative No. patients | Cumulative ICU survivors | Cumulative ICU deaths | Proportion survivors | Proportion deaths | Difference of proportions (95%CI) | Cumulative No. patients | Cumulative ICU survivors | Cumulative ICU deaths | Proportion survivors | Proportion deaths | Difference of proportions (95%CI) |
| **1** | 0 | 0 | 0 | 0 | 0 | - | 0 | 0 | 0 | 0 | 0 | - |
| **2** | 4 | 1 | 3 | 25.0 | 75.0 | -50 (-100 to 35.0) | 2 | 0 | 2 | 0 | 100,0 | -100 (-100 to -50) |
| **3** | 13 | 6 | 7 | 46.2 | 53.9 | -7.7 (-53.7 to 38.3) | 5 | 3 | 2 | 60,0 | 40,0 | 20.0 (-60.7 to 100) |
| **4** | 16 | 7 | 9 | 43.8 | 56.3 | -12.5 (-53.1 to 28.1) | 12 | 8 | 4 | 66,7 | 33,3 | 33.3 (-12.7 to 79.4) |
| **5** | 20 | 10 | 10 | 50.0 | 50.0 | 0 (-31.0 to 31.0) | 23 | 17 | 6 | 73,9 | 26,1 | 47.8 (18.1 to 77.6) |
| **6** | 28 | 16 | 12 | 57.1 | 42.9 | 14.3 (-15.2 to 43.8) | 33 | 26 | 7 | 78,8 | 21,2 | 57.6 (34.8 to 80.3) |
| **7** | 29 | 16 | 13 | 55.2 | 44.8 | 10.3 (-18.7 to 39.4) | 44 | 36 | 8 | 81,8 | 18,2 | 63.6 (45.3 to 82.0) |
| **8** | 34 | 21 | 13 | 61.8 | 38.2 | 23.5 (-2.5 to 49.6) | 54 | 45 | 9 | 83,3 | 16,7 | 66.7 (50.8 to 82.6) |
| **9** | 35 | 21 | 14 | 60.0 | 40.0 | 20.0 (-5.8 to 45.8) | 65 | 53 | 12 | 81,5 | 18,5 | 63.1 (48.2 to 78.0) |
| **10** | 40 | 24 | 16 | 60.0 | 40.0 | 20.0 (-4.0 to 44.0) | 73 | 61 | 12 | 83,6 | 16,4 | 67.1 (53.7 to 80.5) |
| **11** | 43 | 25 | 18 | 58.1 | 41.9 | 16.3 (-6.9 to 39.5) | 80 | 66 | 14 | 82,5 | 17,5 | 65.0 (52.0 to 78.0) |
| **12** | 50 | 28 | 22 | 56.0 | 44.0 | 12.0 (-9.5 to 33.5) | 85 | 70 | 15 | 82,4 | 17,7 | 64.7 (52.1 to 77.3) |
| **13** | 58 | 34 | 24 | 58.6 | 41.4 | 17.2 (-2.4 to 36.9) | 91 | 76 | 15 | 83,5 | 16,5 | 67.0 (55.2 to 78.9) |
| **14** | 61 | 36 | 25 | 59.0 | 41.0 | 18.0 (-1.1 to 37.1) | 95 | 79 | 16 | 83,2 | 16,8 | 66.3 (54.6 to 78.0) |
| **15** | 70 | 41 | 29 | 58.6 | 41.4 | 17.1 (-0.6 to 34.9) | 97 | 79 | 18 | 81,4 | 18,6 | 62.9 (50.9 to 74.9) |
| **16** | 78 | 48 | 30 | 61.5 | 38.5 | 23.1 (6.5 to 39.6) | 102 | 83 | 19 | 81,4 | 18,6 | 62.8 (51.1 to 74.4) |
| **17** | 81 | 49 | 32 | 60.5 | 39.5 | 21.0 (4.7 to 37.3) | 105 | 85 | 20 | 81.0 | 19,1 | 61.9 (50.3 to 73.5) |
| **18** | 85 | 53 | 32 | 62.4 | 37.7 | 24.7 (9.0 to 40.5) | 107 | 87 | 20 | 81,3 | 18,7 | 62.6 (51.2 to 74.0) |
| **19** | 85 | 53 | 32 | 62.4 | 37.7 | 24.7 (9.0 to 40.5) | 110 | 90 | 20 | 81,8 | 18,2 | 63.6 (52.5 to 74.7) |
| **20** | 87 | 55 | 32 | 63.2 | 36.8 | 26.4 (11.0 to 41.9) | 112 | 91 | 21 | 81,3 | 18,8 | 62.5 (51.4 to 73.6) |
| **21** | 90 | 57 | 33 | 63.3 | 36.7 | 26.7 (11.5 to 41.9) | 114 | 91 | 23 | 79,8 | 20,2 | 59.7 (48.4 to 70.9) |
| **22** | 94 | 59 | 35 | 62.8 | 37.2 | 25.5 (10.7 to 40.4) | 115 | 91 | 24 | 79,1 | 20,9 | 58.3 (46.9 to 69.6) |
| **23** | 97 | 62 | 35 | 63.9 | 36.1 | 27.8 (13.3 to 42.4) | 115 | 91 | 24 | 79,1 | 20,9 | 58.3 (46.9 to 69.6) |
| **24** | 99 | 63 | 36 | 63.6 | 36.4 | 27.3 (12.9 to 41.7) | 115 | 91 | 24 | 79,1 | 20,9 | 58.3 (46.9 to 69.6) |
| **25** | 102 | 65 | 37 | 63.7 | 36.3 | 27.5 (13.3 to 41.6) | 115 | 91 | 24 | 79,1 | 20,9 | 58.3 (46.9 to 69.6) |
| **26** | 105 | 67 | 38 | 63.8 | 36.2 | 27.6 (13.7 to 41.6) | 117 | 93 | 24 | 79,5 | 20,5 | 59.0 (47.8 to 70.2) |
| **27** | 111 | 73 | 38 | 65.8 | 34.2 | 31.5 (18.2 to 44.9) | 119 | 94 | 25 | 79.0 | 21,0 | 58.0 (46.8 to 69.2) |
| **28** | 114 | 76 | 38 | 66.7 | 33.3 | 33.3 (20.2 to 46.5) | 119 | 94 | 25 | 79.0 | 21,0 | 58.0 (46.8 to 69.2) |
| **29** | 115 | 76 | 39 | 66.1 | 33.9 | 32.2 (19.1 to 45.3) | 119 | 94 | 25 | 79.0 | 21,0 | 58.0 (46.8 to 69.2) |
| **30** | 115 | 76 | 39 | 66.1 | 33.9 | 32.2 (19.1 to 45.3) | 123 | 98 | 25 | 79,7 | 20,3 | 59.4 (48.5 to 70.2) |
| **31** | 115 | 76 | 39 | 66.1 | 33.9 | 32.2 (19.1 to 45.3) | 124 | 99 | 25 | 79,8 | 20,2 | 59.7 (48.9 to 70.5) |
| **32** | 115 | 76 | 39 | 66.1 | 33.9 | 32.2 (19.1 to 45.3) | 126 | 101 | 25 | 80,2 | 19,8 | 60.3 (49.7 to 71.0) |
| **33** | 119 | 79 | 40 | 66.4 | 33.6 | 32.8 (19.9 to 45.6) | 129 | 104 | 25 | 80,6 | 19,4 | 61.2 (50.8 to 71.7) |
| **34** | 119 | 79 | 40 | 66.4 | 33.6 | 32.8 (19.9 to 45.6) | 130 | 105 | 25 | 80,8 | 19,2 | 61.5 (51.2 to 71.9) |
| **35** | 121 | 81 | 40 | 66.9 | 33.1 | 33.9 (21.2 to 46.6) | 130 | 105 | 25 | 80,8 | 19,2 | 61.5 (51.2 to 71.9) |
| **36** | 122 | 82 | 40 | 67.2 | 32.8 | 34.4 (21.8 to 47.0) | 131 | 106 | 25 | 80,9 | 19,1 | 61.8 (51.6 to 72.1) |
| **37** | 122 | 82 | 40 | 67.2 | 32.8 | 34.4 (21.8 to 47.0) | 131 | 106 | 25 | 80,9 | 19,1 | 61.8 (51.6 to 72.1) |
| **38** | 124 | 83 | 41 | 66.9 | 33.1 | 33.9 (21.4 to 46.4) | 131 | 106 | 25 | 80,9 | 19,1 | 61.8 (51.6 to 72.1) |
| **39** | 124 | 83 | 41 | 66.9 | 33.1 | 33.9 (21.4 to 46.4) | 131 | 106 | 25 | 80,9 | 19,1 | 61.8 (51.6 to 72.1) |
| **40** | 126 | 85 | 41 | 67.5 | 32.5 | 34.9 (22.6 to 47.3) | 131 | 106 | 25 | 80,9 | 19,1 | 61.8 (51.6 to 72.1) |
| **41** | 128 | 87 | 41 | 68.0 | 32.0 | 35.9 (23.7 to 48.2) | 131 | 106 | 25 | 80,9 | 19,1 | 61.8 (51.6 to 72.1) |
| **42** | 129 | 88 | 41 | 68.2 | 31.8 | 36.4 (24.3 to 48.6) | 132 | 107 | 25 | 81,1 | 18,9 | 62.1 (51.9 to 72.3) |
| **43** | 130 | 89 | 41 | 68.5 | 31.5 | 36.9 (24.9 to 49.0) | 132 | 107 | 25 | 81,1 | 18,9 | 62.1 (51.9 to 72.3) |
| **44** | 130 | 89 | 41 | 68.5 | 31.5 | 36.9 (24.9 to 49.0) | 132 | 107 | 25 | 81,1 | 18,9 | 62.1 (51.9 to 72.3) |
| **45** | 132 | 91 | 41 | 68.9 | 31.1 | 37.9 (26.0 to 49.8) | 132 | 107 | 25 | 81,1 | 18,9 | 62.1 (51.9 to 72.3) |
| **46** | 132 | 91 | 41 | 68,9 | 31,1 | 37.9 (26.0 to 49.8) | 132 | 107 | 25 | 81,1 | 18,9 | 62.1 (51.9 to 72.3) |
| **47** | 132 | 91 | 41 | 68,9 | 31,1 | 37.9 (26.0 to 49.8) | 132 | 107 | 25 | 81,1 | 18,9 | 62.1 (51.9 to 72.3) |
| **48** | 132 | 91 | 41 | 68,9 | 31,1 | 37.9 (26.0 to 49.8) | 134 | 108 | 26 | 80,6 | 19,4 | 61.2 (51.0 to 71.4) |
| **49** | 132 | 91 | 41 | 68,9 | 31,1 | 37.9 (26.0 to 49.8) | 134 | 108 | 26 | 80,6 | 19,4 | 61.2 (51.0 to 71.4) |
| **50** | 132 | 91 | 41 | 68,9 | 31,1 | 37.9 (26.0 to 49.8) | 135 | 109 | 26 | 80,7 | 19,3 | 61.5 (51.3 to 71.6) |
| **51** | 133 | 91 | 42 | 68,4 | 31,6 | 36.8 (24.9 to 48.8) | 135 | 109 | 26 | 80,7 | 19,3 | 61.5 (51.3 to 71.6) |
| **52** | 134 | 92 | 42 | 68,7 | 31,3 | 37.3 (25.5 to 49.2) | 135 | 109 | 26 | 80,7 | 19,3 | 61.5 (51.3 to 71.6) |
| **53** | 136 | 94 | 42 | 69,1 | 30,9 | 38.2 (26.5 to 50.0) | 136 | 110 | 26 | 80,9 | 19,1 | 61.8 (51.7 to 71.9) |
| **54** | 136 | 94 | 42 | 69,1 | 30,9 | 38.2 (26.5 to 50.0) | 136 | 110 | 26 | 80,9 | 19,1 | 61.8 (51.7 to 71.9) |
| **55** | 136 | 94 | 42 | 69,1 | 30,9 | 38.2 (26.5 to 50.0) | 136 | 110 | 26 | 80,9 | 19,1 | 61.8 (51.7 to 71.9) |
| **56** | 136 | 94 | 42 | 69,1 | 30,9 | 38.2 (26.5 to 50.0) | 136 | 110 | 26 | 80,9 | 19,1 | 61.8 (51.7 to 71.9) |
| **57** | 136 | 94 | 42 | 69,1 | 30,9 | 38.2 (26.5 to 50.0) | 136 | 110 | 26 | 80,9 | 19,1 | 61.8 (51.7 to 71.9) |
| **58** | 136 | 94 | 42 | 69,1 | 30,9 | 38.2 (26.5 to 50.0) | 136 | 110 | 26 | 80,9 | 19,1 | 61.8 (51.7 to 71.9) |
| **59** | 136 | 94 | 42 | 69,1 | 30,9 | 38.2 (26.5 to 50.0) | 137 | 111 | 26 | 81,0 | 19.0 | 62.0 (52.0 to 72.1) |
| **60** | 136 | 94 | 42 | 69,1 | 30,9 | 38.2 (26.5 to 50.0) | 138 | 112 | 26 | 81,2 | 18,8 | 62.3 (52.4 to 72.3) |
| **>60** | 138 | 95 | 43 | 68,8 | 31,2 | 37.7 (26.0 to 49.3) | 139 | 113 | 26 | 81,3 | 18,7 | 62.6 (52.7 to 72.5) |

*ARDS: acute respiratory distress syndrome; CI: confidence interval; ICU: intensive care unit; No.: number.*

**TABLE S7. Baseline characteristics (at the time of meeting criteria for moderate-to-severe acute respiratory distress syndrome) and outcome data of 277 patients included in the DEXA-ARDS trial at ICU discharge.**

| **Variables** | **All patients (N=277)** | **Survivors**  **N= 208** | **Non-survivors**  **N= 69** | **Mean difference (95%CI)** | **p-value** |
| --- | --- | --- | --- | --- | --- |
| Age, *years, mean±SD* | 57.1±14.5 | 54.6±13.6 | 64.9±14.3 | 10.3 (6.5 to 14.1) | <0.001 |
| Sex, *n (%)*  Male  Female | 191 (69.0)  86 (31.0) | 140 (67.3)  68 (32.7) | 51 (73.9)  18 (26.1) | 6.6 (-6.3 to 17.7) | 0.305 |
| Etiology*, n (%)*  Pneumonia  Sepsis  Aspiration  Trauma  Acute Pancreatitis  Multiple transfusions  Others | 147 (53.1)  59 (21.3)  33 (11.9)  21 (7.6)  8 (2.9)  5 (1.8)  4 (1.4) | 118 (56.7)  44 (21.2)  16 (7.7)  20 (9.6)  4 (1.9)  3 (1.4)  3 (1.4) | 29 (42.0)  15 (21.7)  17 (24.6)  1 (1.4)  4 (5.8)  2 (2.9)  1 (1.4) | 14.7 (1.1 to 27.4)  0.5 (-9.6 to 12.6)  16.9 (7.2 to 28.6)  8.2 (1.1 to 13.1)  3.9 (-0.7 to 12.2)  1.5 (-1.9 to 8.6)  0.0 (-6.4 to 2.9) | 0.034  0.930  <0.001  0.026  0.093  0.414  1.0 |
| Degree of ARDS, *n (%)*  Severe  Moderate | 100 (36.1)  177 (63.9) | 77 (37.0)  131 (63.0) | 23 (33.3)  46 (66.7) | 3.7 (-9.6 to 15.7) | 0.580 |
| APACHE II score, *mean±SD* | 19.0±7.0 (**§**) | 18.0±6.8 | 22.0±7.0 | 4.0 (2.1 to 5.9) | <0.001 |
| SOFA score, *mean±SD* | 8.8±3.0 | 8.5±3.0 | 9.8±3.0 | 1.3 (0.5 to 2.1) | 0.002 |
| PaO_2_/FiO_2_, *mmHg, mean±SD* | 118.9±37.6 | 118.8±37.9 | 119.3±37.0 | 0.5 (-9.8 to 10.8) | 0.924 |
| FiO_2_, *mean±SD* | 0.78±0.19 | 0.79±0.19 | 0.75±0.19 | 0.04 (-0.01 - 0.09) | 0.131 |
| PaO_2_, *mmHg, mean±SD* | 88.6±26.9 | 89.9±28.1 | 84.9±22.7 | 5.0 (-2.3 to 12.3) | 0.182 |
| PaCO_2_, *mmHg, mean±SD* | 49.6±11.7 | 49.1±11.8 | 51.1±11.3 | 2.0 (-1.2 to 5.2) | 0.219 |
| pH, *mean±SD* | 7.31±0.10 | 7.32±0.10 | 7.29±0.11 | 0.03 (0.00 to 0.06) | 0.036 |
| VT, *mL/kg PBW, mean±SD* | 7.1±1.1 | 7.2±1.1 | 7.1±1.0 | 0.1 (-0.4 to 0.2) | 0.504 |
| Respiratory rate, *mean±SD* | 21.4±4.2 | 21.3±4.1 | 21.8±4.5 | 0.5 (-1.6 to 0.6) | 0.393 |
| Minute ventilation, *L/min,* *mean±SD* | 9.6±2.0 | 9.7±2.0 | 9.4±2.1 | 0.3 (-0.3 to 0.9) | 0.287 |
| PEEP, cmH_2_O, *mean±SD* | 12.0±3.3 | 12.1±3.4 | 11.7±3.1 | 0.4 (-1.3 to 0.5) | 0.388 |
| Plateau pressure, *cmH_2_O, mean±SD* | 26.5±4.4 (**¶**) | 26.7±4.3 | 25.9±4.7 | 0.8 (-0.4 to 2.0) | 0.192 |
| Driving pressure, *cmH_2_O,* *mean±SD* | 14.5±4.0 | 14.7±3.8 | 14.1±4.7 | 0.6 (-0.5 to 1.7) | 0.286 |
| No. extrapulmonary OF, *mean±SD* | 1.6±1.1 | 1.5±1.0 | 1.9±1.2 | 0.4 (0.1 to 0.7) | 0.007 |
| Days from initiation of MV to diagnosis of moderate/severe ARDS, *d, mean±SD* | 1.0±2.6 | 1.0±2.7 | 1.0±2.1 | 0 (-0.7 to 0.7) | 0.911 |
| Days ventilatory support from diagnosis of moderate/severe ARDS, *d, mean±SD* | 16.8±13.4 | 17.5±13.8 | 14.4±11.9 | 3.1 (-0.6 to 6.8) | 0.096 |
| All-cause ICI mortality, *n (%)* | 69 (24.9) | - | - | - | - |
| All-cause hospital mortality, *n (%)* | 83 (30.0) | - | - | 5.1 (-2.3 to 12.5) | 0.179 |

*APACHE: acute physiology and chronic health evaluation; ARDS: acute respiratory distress syndrome; CI: confidence interval; d: days; FiO_2_: fraction of inspired oxygen concentration; ICU: intensive care unit; OF: organ failure; PBW: predicted body weight; PEEP: positive end-expiratory pressure; SD: standard deviation; SOFA: sequential organ failure assessment scale; VT: tidal volume.*

*(§) APACHE II was missing in 1 survivor.*

*(¶) Plateau pressure was missing in 3 survivors and 4 non-survivors.*

**SUPPLEMENTARY REFERENCES**

1. World Medical Association. World Medical Association Declaration of Helsinki: Ethical Principles for Medical Research Involving Human Subjects. *JAMA* 2013; 310:2191-2194
2. von Elm E, Altman DG, Egger M, Pocock SJ, Gøtzsche PC, Vandenbroucke JP for the STROBE Initiative. The strengthening the reporting of observational studies in epidemiology (STROBE) statement: guidelines for reporting observational studies. *PLoS Medicine* 2007; 4:e296
3. Villar J, Blanco J, Añón JM, Santos-Bouza A, Blanch L, Ambrós A, et al. The ALIEN study: Incidence and outcome of acute respiratory distress syndrome in the era of lung protective ventilation. *Intensive Care Med* 2011; 37:1932-1941
4. Villar J, Pérez-Méndez, Blanco J, Añón JM, Blanch L, Belda J, et al; Spanish Initiative for Epidemiology, Stratification, and Therapies for ARDS (SIESTA) Network. A universal definition of ARDS: the PaO_2_/FiO_2_ ratio under a standard ventilatory setting – a prospective, multicenter validation study. *Intensive Care Med* 2013; 39:583-592
5. Villar J, Blanco J, del Campo R, Andaluz-Ojeda D. Díaz Dominguez FJ, Muriel A, et al. Assessment of PaO2/FiO2 for stratification of patients with moderate and severe acute respiratory distress syndrome. *BMJ Open* 2015; 5:1006812
6. Villar J, Mora-Ordoñez JM, Soler JA, Mosteiro F, Vidal A, Ambrós A, et al. The PANDORA study: Prevalence and outcome of acute hypoxemic respiratory failure in the pre-COVID era. *Crit Care Explor* 2022; 4:e0684
7. Ranieri VM, Rubenfeld GD, Thompson BT, Ferguson ND, Caldwell E, Fan E, et al. Acute respiratory distress syndrome: The Berlin definition. *JAMA* 2012; 307:2526-2533
8. Villar J, Ambrós A, Soler JA, Martínez D, Ferrando C, Solano R, et al. Age, PaO_2_/FiO_2_, and plateau pressure score: a proposal for a simple outcome score in patients with the acute respiratory distress syndrome. *Crit Care Med* 2016; 44:1361-1369
9. Villar J, Martín-Rodríguez C, Domínguez-Berrot AM, Fernández L, Ferrando C, Soler JA, et al. A quantile analysis of plateau and driving pressures: Effects on mortality in patients with acute respiratory distress syndrome receiving lung-protective ventilation. *Crit Care Med* 2017; 45:843-850
10. Villar J, Ambrós A, Mosteiro F, Martínez D, Fernández L, Ferrando C, et al. A prognostic enrichment strategy for selection of patients with acute respiratory distress syndrome in clinical trials. *Crit Care Med* 2019; 47:377-385
11. Villar J, González-Martín JM, Ambrós A, Mosteiro F, Martínez D, Fernández L, et al; Spanish Initiative for Epidemiology, Stratification and Therapies of ARDS (SIESTA) Network. Stratification for Identification of Prognostic Categories In the Acute RESpiratory Distress Syndrome (SPIRES) Score. *Crit Care Med* 2021; 49:e920-e930
12. Villar J, Ferrando C, Martínez D, Ambrós A, Muñoz T, Soler JA, et al; dexamethasone in ARDS network. Dexamethasone treatment for the acute respiratory distress syndrome: a multicentre, randomised controlled trial. *Lancet Respir Med* 2020; 8:267-276
13. Bernard GR, Artigas A, Brigham KL, Carlet J, Falke K, Hudson L, et al. The American-European Consensus Conference on ARDS. Definitions, mechanisms, relevant outcomes, and clinical trial coordination. *Am J Respir Crit Care Med* 1994; 149:818-824
14. Guerin C, Thompson T, Brower R. The ten diseases that look like ARDS. *Intensive Care Med* 2015; 41:1099-1102
15. Acute Respiratory Distress Syndrome Network. Ventilation with lower tidal volumes as compared with traditional tidal volumes for acute lung injury and the acute respiratory distress syndrome. *N Engl J Med* 2000; 342:1301-1308
16. Detsky AS. Learning the art and science of diagnosis. *JAMA* 2022; 327:1759-1760
17. Bellani G, Laffey JG, Pham T, Fan E, Brochard L, Esteban A, et al. Epidemiology, patterns of care, and mortality for patients with acute respiratory distress syndrome in intensive care units in 50 countries. *JAMA* 2016; 315:788-800

**ACKNOWLEDGEMEMTS**

**Members of the Spanish Initiative for Epidemiology, Stratification and Therapies of ARDS (SIESTA) Network are listed below:**

- Jesús Villar, Rosa L. Fernández, Cristina Fernández, Jesús M. González-Martin, Pedro Rodríguez-Suárez (Hospital Universitario Dr. Negrín, Las Palmas de Gran Canaria, Spain);
- Alfonso Ambrós, Rafael del Campo, Carmen Martín-Rodríguez, Ana Bueno-González, Carmen Hornos-López (Hospital General Universitario, Ciudad Real, Spain);
- Fernando Mosteiro, Ana M. Díaz-Lamas, Regina Arrojo, Lidia Pita-García (Complejo Hospitalario Universitario de La Coruña, La Coruña, Spain);
- Lorena Fernández, Jesús Sánchez-Ballesteros, Jesús Blanco, Arturo Muriel, Pablo Blanco-Schweizer, José Ángel de Ayala, César Aldecoa, Jesús Rico-Feijoo, Alba Pérez, Silvia Martín-Alfonso (Hospital Universitario Río Hortega, Valladolid, Spain);
- Domingo Martínez, Juan A. Soler, Ana M. del Saz-Ortiz, Luís A. Conesa-Cayuela (Hospital Universitario Virgen de Arrixaca, Murcia, Spain);
- Demetrio Carriedo, Ana M. Domínguez-Berrot, Francisco J. Díaz-Domínguez, Raúl I. González-Luengo (Complejo Hospitalario Universitario de León, León, Spain);
- Lucia Capilla (Hospital General Universitario Rafael Méndez, Lorca, Murcia, Spain);
- David Andaluz, Leonor Nogales, Laura Parra (Hospital Clínico Universitario, Valladolid, Spain);
- Elena González-Higueras, Rosario Solano, María J. Bruscas (Hospital Virgen de la Luz, Cuenca, Spain);
- Blanca Arocas, Marina Soro, Javier Belda, Andrea Gutiérrez, Ernesto Pastor, Gerardo Aguilar (Hospital Clínico Universitario, Valencia, Spain);
- Carlos Ferrando (Hospital Clinic, Barcelona, Spain);
- José M. Añón, Belén Civantos, Mónica Hernández (Hospital Universitario La Paz, Madrid, Spain);
- Raquel Montiel, Dácil Parrilla, Eduardo Peinado, Lina Pérez-Méndez (Hospital Universitario NS de Candelaria, Tenerife, Spain);
- Anxela Vidal, Denis Robaglia, César Pérez (Hospital Universitario Fundación Jiménez Díaz, Madrid, Spain);
- María del Mar Fernández (Hospital Universitario Mutua Terrassa, Terrassa, Barcelona, Spain);
- Eleuterio Merayo, Chanel Martínez-Jiménez, Ángeles de Celis-Álvarez (Hospital del Bierzo, Ponferrada, León, Spain);
- Juan M. Mora-Ordoñez, J. Francisco Martínez-Carmona, Álvaro Valverde-Monto, Victoria Olea-Jiménez (Hospital Regional Universitario de Málaga, Málaga, Spain);
- Concepción Tarancón, Silvia Cortés-Díaz (Hospital Virgen de la Concha, Zamora, Spain);
- Carmen Martín-Delgado (Hospital La Mancha Centro, Alcázar de San Juan, Ciudad Real, Spain);
- Francisca Prieto (Hospital Santa Bárbara, Puertollano, Ciudad Real, Spain);
- Isidro Prieto, Mario Chico, Darío Toral (Hospital Universitario 12 de Octubre, Madrid, Spain);
- Miguel A. Romera, Carlos Chamorro-Jambrina (Hospital Universitario Puerta de Hierro, Majadahonda, Madrid, Spain);
- Alec Tallet, Santiago Macías, Noelia Lázaro (Hospital General de Segovia, Segovia, Spain);
- Isabel Murcia, Ángel E. Pereyra (Hospital General Universitario de Albacete, Albacete, Spain);
- Francisco Alba, Ruth Corpas (Hospital NS del Prado, Talavera de la Reina, Toledo, Spain);
- David Pestaña, Pilar Cobeta, Adrián Mira (Hospital Universitario Ramón y Cajal, Madrid, Spain);
- Francisca Prieto (Hospital Santa Barbara, Puertollano, Ciudad Real, Spain);
- Lluis Blanch, Gemma Gomá, Gisela Pili (Corporació Sanitaria Parc Taulí, Sabadell, Barcelona, Spain);
- Antonio Santos-Bouza, Cristina Domínguez (Complejo Hospitalario Universitario de Santiago, Santiago de Compostela, La Coruña, Spain);
- Javier Collado, José I. Alonso (Hospital Río Carrión, Palencia, Spain);
- Alberto Indarte, María E. Perea (Hospital General Yagüe, Burgos, Spain);
- Ricardo Fernández, José I. Lozano (Hospital de Hellín, Albacete, Spain);
- Robert M. Kacmarek *(deceased)* (Massachussets General Hospital, Boston, Massachusetts, USA);
- Tamas Szakmany (Department of Intensive Care Medicine & Anesthesia, Aneurin Bevan University Health Board, Newport, Wales, United Kingdom);
- Karen E.A. Burns (Department of Critical Care Medicine, Unity Health Toronto-St. Michael’s Hospital, Toronto, Canada);
- Ewout W. Steyerberg (Department of Biomedical Data Sciences, Leiden University Medical Center, Leiden, The Netherlands);
- Arthur S. Slutsky (Li Ka Shing Knowledge Institute, St. Michael’s Hospital, Toronto, Ontario, Canada).
